# Mapping the Molecular Landscape of Human DLBCL by GCIB-SIMS

**DOI:** 10.1021/acs.analchem.4c06594

**Published:** 2025-03-26

**Authors:** Simon Uzoni, Daniele Zanchin, Vasilis Chatzikyriakos, Noora Neittaanmäki, John S. Fletcher

**Affiliations:** † Department of Chemical and Molecular Biology, University of Gothenburg, 413 90 Gothenburg, Sweden; ‡ Department of Laboratory Medicine, Institute of Biomedicine, Sahlgrenska Academy, University of Gothenburg, 413 45 Gothenburg, Sweden; § Department of Clinical Pathology and Cytology, Sahlgrenska University Hospital, Region Västra Götaland, 413 45 Gothenburg, Sweden

## Abstract

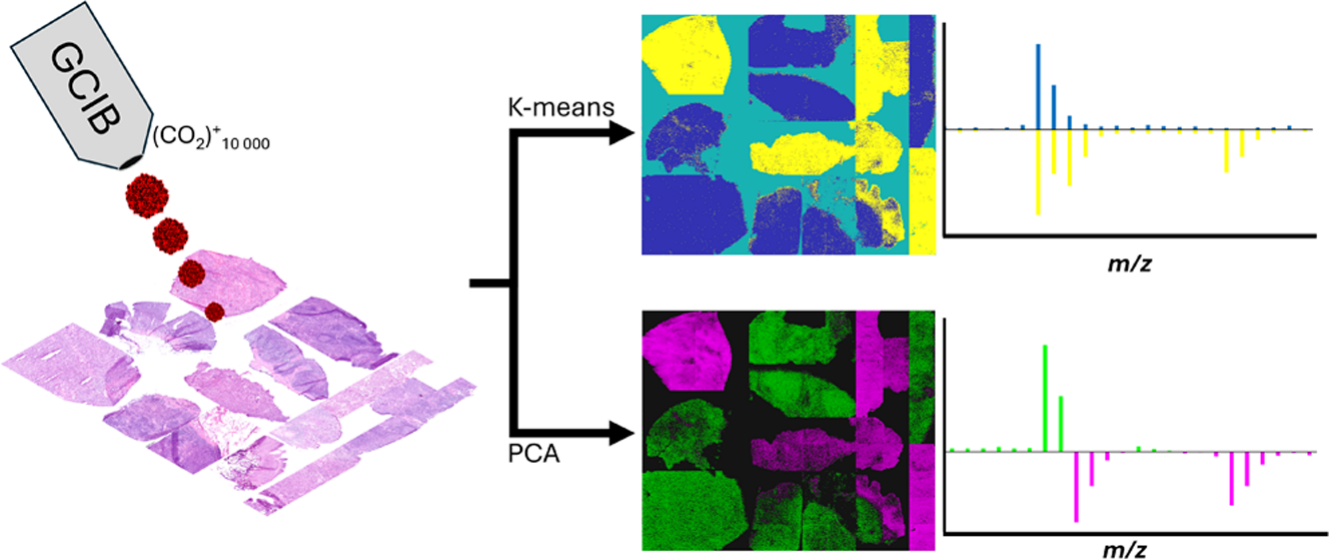

Secondary ion mass spectrometry (SIMS) using a gas cluster ion beam (GCIB), in this case 40 keV (CO_2_)_7k^+^_, was used to map the intact lipid signals across 14 lymph node samples representing diffuse large B-cell lymphoma (DLBCL), a common and aggressive form of lymphoma, and nonmalignant controls. The analysis allowed the samples to be classified as malignant or nonmalignant and also highlighted additional aggressive cancer signature in a DLBCL sample with an unusually high proliferation index. A complementary, combined *k*-means/image PCA approach was used to interrogate the data, highlighting the pros and cons of the different approaches and potential sources for misclassification/diagnoses resulting from the heterogeneity of the DLBCL samples. Compared to other cancer types, lymphoma results in a reduction of non-neoplastic inflammatory cells and their characteristic signals that are often classed as cancer-related, highlighting the need to consider disease heterogeneity when examining MS data. While delivering new information regarding the chemistry of lymphoma, the results also highlight the need for cellular precision with high chemical specificity and sensitivity, and the challenges associated with spectral/spatial classification of such complex samples and data where differently aggressive cancer samples show different signatures and pockets of different cell types, in this case histiocytes, can show intermediate cancer/healthy lipid profiles.

## Introduction

Lymphomas are a diverse group of malignant neoplasms with lymphoid-cell origin and represent one of the most common types of cancer.^1^ Lymphomas represent 4% of the malignancies in Western countries, making them the fifth most common category of cancer and thus cause of cancer mortality.^1−3^ The survival rates of different subtypes of lymphomas vary significantly, but the combined 5-year overall survival is estimated to be about 70%.^2^ Despite lymphomas being broadly classified as Hodgkin lymphoma (HL) and non-Hodgkin lymphoma (NHL), the World Health Organization (WHO) has recognized approximately 70 different types of lymphomas and divided them into four broad groups: mature B-cell neoplasm, mature T-cell and natural killer cell neoplasm, Hodgkin lymphoma, and immunodeficiency-associated lymphoproliferative disorders.

Lymphomas derived from B cells represent more than 80% of lymphomas, diffuse large B-cell lymphoma (DLBCL) being the most common type of NHL, accounting for approximately 20–25% of all lymphomas among adults in the Western world.^3^ The disease is aggressive, and patients typically present with rapidly enlarging lymphadenopathy necessitating immediate treatment. Although most patients present with lymphadenopathy, there is also a high frequency, approximately 40%, with rapidly growing tumors in spleen, liver, bone marrow, or other organs.^4^ The most common frontline treatment is a combination of immunotherapy and chemotherapy which leads to a cure in approximately 50–60% of patients. The remaining patients with refractory disease to frontline treatment or relapse after achieving remission have particularly poor outcomes, achieving long-term remissions in only a minority of patients with high-dose chemotherapy and autologous stem cell transplantation.^5^

The most critical step in diagnosing lymphoma is to establish an accurate histological diagnosis by obtaining tumor samples from the lymph nodes or other organs. While histological assessment of tissue samples remains the foundation of the lymphoma diagnosis, pathologists are increasingly turning to newer techniques like cytogenetic testing, fluorescence in situ hybridization (FISH), DNA amplification by polymerase chain reaction (PCR), and next-generation sequencing (NGS) to complement the decision-making process.^1^ An understanding of the molecular alterations that characterize both the neoplastic cells and their microenvironment have led to therapeutic advances, targeting both neoplastic and reactive components.^1^

Imaging mass spectrometry (also known as mass spectrometry imaging, MSI) provides a potentially powerful tool for probing the localized chemistry of the tumor microenvironment.^6^ Secondary ion mass spectrometry (SIMS) uses a focused ion beam to eject material from a sample surface for analysis with mass spectrometry. The ion beams are relatively easy to focus providing spot sizes of tens of nanometers to several micrometers and require minimal sample preparation/pretreatment.^7^ A drawback of SIMS for biological analysis has been the poor sensitivity to larger, intact, molecular ions from a sample; however, advances in ion beam technology have facilitated a shift to routine detection of molecular species up to several kDa. This increase in molecular detection arises from the use of gas cluster ion beams (GCIBs) for analysis where the energy of the impinging ion is distributed over several thousand constituent atoms of molecules.^8^ Clusters of Ar, CO_2_, and H_2_O have been used to analyze various cell and tissue samples with the demonstration of the potential for peptide and protein fragment analysis also reported.^9−11^

In this study, we employ imaging SIMS using a CO_2_ GCIB to elucidate the chemical changes in human DLBCL that may lead to improved diagnostics and treatment strategies. Image analysis using complementary multivariate analysis (MVA) methods revealed general markers for the classification of healthy versus cancerous tissue and also intriguing insights into inflammatory cell variation along with chemical signals related to hyperproliferation and cellular turnover in an especially aggressive tumor.

## Materials and Methods

### Tissue Preparation

Analysis was performed on a pilot cohort set consisting of five lymph nodes representing DLBCL and seven samples representing reactive nonmalignant lymph nodes that, for simplicity in this manuscript, will be referred to as “cancerous/cancer” and “healthy”, respectively. The material was from fresh frozen tissue samples stored at the Department of Pathology, Sahlgrenska University Hospital. The ethical approval was granted by the ethics committee (Dnr 2021-02385)

The samples were sliced 6 μm thick using a cryomicrotome (Leica CM 3050S) and thaw-mounted onto indium tin oxide (ITO)-coated glass slides (Bruker Daltonik GmbH, Germany).

Consecutive tissue sections to every section used for SIMS analysis were stained with hematoxylin and eosin (H&E) and evaluated by a pathologist (VC) with routine light microscopy. Furthermore, we had access to the original histopathological diagnostic information and the glass slides representing the paraffin-embedded formalin-fixed material and the immunohistochemical slides for each case. Samples were transported on dry ice to the SIMS laboratory and stored at −80 °C. Prior to SIMS analysis, the samples were mounted onto a precooled (−20 °C) copper sample holder under nitrogen gas before being transferred to the SIMS instrument and allowed to warm up under vacuum in the instrument’s preparation chamber.

### SIMS Analysis

SIMS analysis was performed on a J105 ToF-SIMS instrument (Ionoptika Ltd., U.K.). Unlike conventional ToF-SIMS instruments, a quasi-continuous primary ion beam is used to produce a stream of secondary ions that are pulsed using a linear buncher into a ToF mass analyzer with a reflectron, effectively decoupling the ionization and MS analysis for improved mass resolution and mass accuracy.^12^ In this study, a 40 keV beam of (CO_2_)_7k^+^_ gas clusters was used for analysis. The clusters were produced by supersonic expansion of CO_2_ in vacuum, electron impact ionization, and size selection using a Wein filter.^13^ Low-energy (12 eV) electron flooding was used for charge compensation. Images were acquired in tiled/mosaic mode using a raster size of 900 μm × 900 μm and a raster resolution of 128 × 128 pixels. The primary ion dose density used was 1–2 × 10^12^ ions/cm^2^.

### Data Processing and Analysis

The data sets, recorded in negative-ion mode, were processed by first calibrating the mass spectrum in each mass spectral image in Image Analyser version 3.3.11 (Ionoptika Ltd., U.K.). From every image, normalized total ion current (TIC) mass spectra were collected and used to create two averaged peak tables, *m*/*z* 590–800 and *m*/*z* 800–1190, for automated and centroided peak picking in MATLAB (R2023b) (MathWorks, Natick, MA) using ChiToolBox,^14^ as described by Fransson et al.^15^ In brief, the peak picking algorithm processes the mass spectra by first applying a Gaussian smoothing, then calculates a second derivate with a smoothing window, positive features are removed, inverts the derivative, detects peak position, and keeps a specified number of the most intense peaks in each mass range. The algorithm combines unresolved peaks due to isobaric interference into the same centroid, which needs to be kept in mind while interpreting the MVA results. MATLAB code and parameters for peak picking are detailed in the Supporting Information. The mass spectral images were converted from the vendor-specific file format to a third-party file format readable by MATLAB. In MATLAB, each image was peak-picked and centroided using the peak tables, and the data from the two mass ranges were combined into a single mass range. The intensity of all peaks was sum-normalized to 1 for each individual pixel and square rooted. All of the images were combined into a single image array for further processing. To focus data analysis on the tissue samples, the signal from the ITO glass slide was removed by zero-filling pixels using a *k*-means (*k* = 2) generated mask. Imaging principal components analysis (PCA) and *k*-means clustering were used to interrogate the large, combined SIMS image.

Both PCA and *k*-means were used due to their complementarity and different ways the results are reported. *k*-means produces clusters where each pixel can only be assigned to one cluster, but the sample variables (peaks) can be used to explain all of the different clusters so identifying unique biomarkers is more difficult.^16^ PCA scores the same pixel on multiple principal components (PCs), but for a specific PC, each variable can only be assigned either positively or negatively. To paraphrase, the *k*-means produces images that are more easily correlated with tissue features, while the loadings from PCA are simpler to interpret. Lipids suggested by PCA and *k*-means to differentiate healthy and cancerous samples were validated by inspecting individual ion images for differences in localization in tissues.

Lipids were putatively assigned based on accurate mass measurement using LIPID MAPS Structure Database^17^ and comparison to published work on lymphoma lipid extracts. Care must be taken with the LIPID MAPS Structure Database as it includes computer-generated structures. Hence, in the case of multiple hits, selection was made based on the presence of existing examples, association with mammalian organisms, and propensity to form negative ions. PS lipids fragment to produce a PA-like ion in SIMS so PA assignments were not made if a significant, intact PS lipid signal was also detected. A table of lipid assignments along with mass accuracy of the assignments and assignment confidence based on recommended metabolomics reporting standards^18^ are included in Table S1. Additionally, a complete list of suggested lipids based on a Lipid Maps search with a selected delta-*m*/*z* of ±0.01 is provided, with the selected lipid highlighted in Table S2. Mean mass accuracy of assignments was 4.8 ppm with 78% of assignments having <5 ppm error. All lipid peaks below *m*/*z* 900 are assigned a metabolomic reporting confidence of 2 while higher mass species that may be fragments of larger gangliosides are considered level 2/3. All reported ions are [M – H]^−^ unless specified otherwise.

## Results and Discussion

### *k*-Means and PCA for Identification of DLBCL

**Figure 1:**
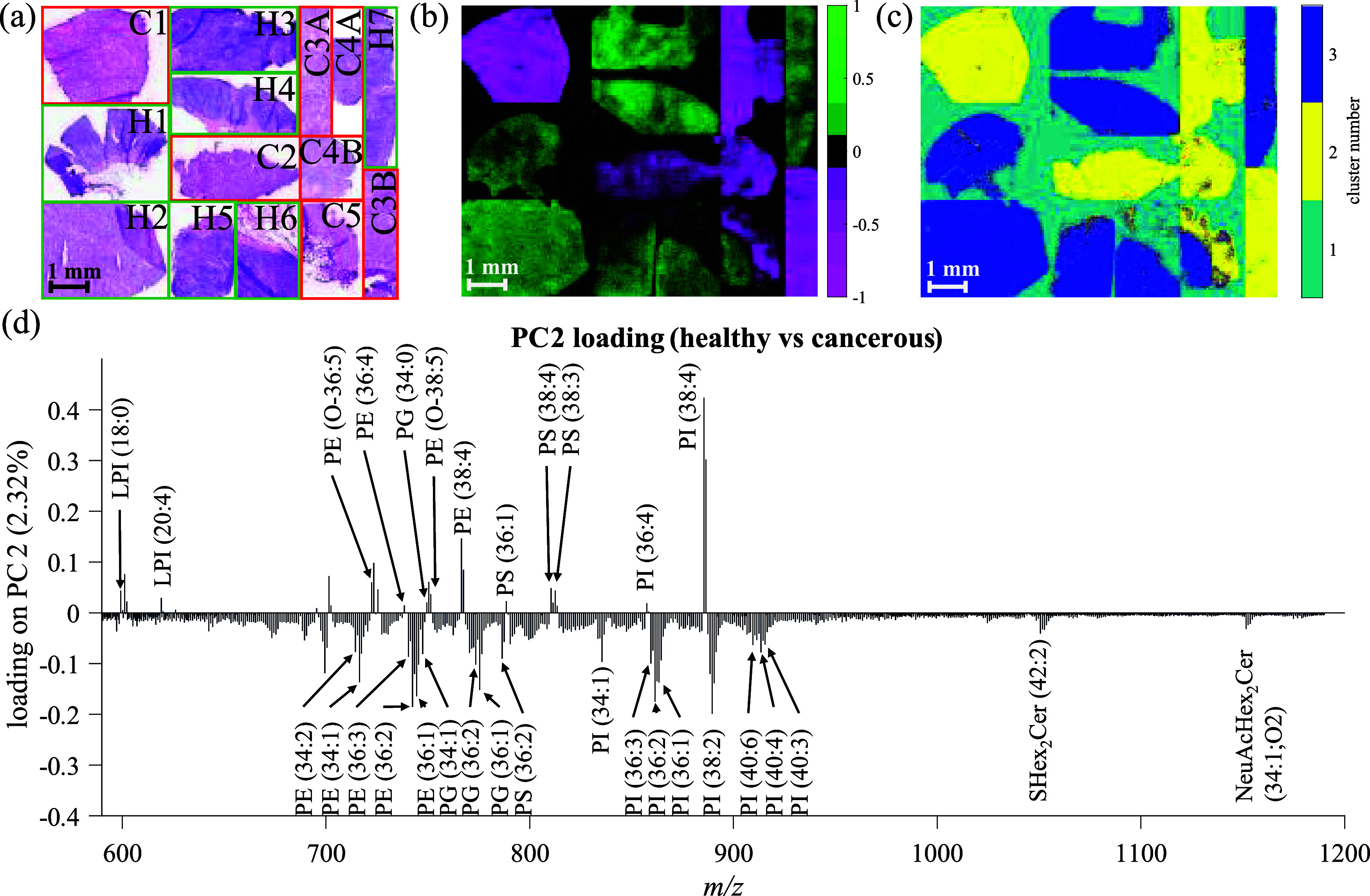
(a) H&E image array of cancerous (C, red frame) and healthy (H, green frame) lymphoid tissues arranged to match (b) PC2 score image and (c) *k*-means image (*k* = 3) results from the SIMS image array. (d) Annotated PC2 loading. Cluster number 1 corresponds to the cancerous tissues, cluster number 2 is the substrate, and cluster number 3 is the healthy tissue. Scale bar: 1 mm. Additional PCA and *k*-means results are available in the Supporting Information.

A composite SIMS image array, total pixel count of 1.62 megapixels, containing the healthy and cancerous tissue images was analyzed by *k*-means clustering and PCA to elucidate differences between cancerous and noncancerous specimens. Prior to both *k*-means and PCA, the ITO-containing regions were detected (using *k*-means), and the signal from these pixels was set to zero. *k*-means clustering requires prespecification of the number of clusters to be generated, so in this initial analysis, 3 clusters were selected to target substrate, normal tissue, and cancerous tissue. Figure 1 shows the results from the *k*-means analysis and the scores image of PC2 (PC1 captured the greatest variance, which was tissue versus zero filled substrate). H&E-stained tissue images show the distribution of tissue samples in the composite image and labeled as cancerous (C) or healthy (H) in Figure 1a. Larger images of the H&E-stained consecutive sections are provided in Figures S1–S14. To target changes in lipid signals and avoid contribution from intense, less specific, fragment ions, the mass range *m*/*z* 590–1190 was used for this data analysis.

Imaging PCA was able to differentiate the tissues into healthy and DLBCL as seen in Figure 1b where the healthy tissues are mostly colored in with green pixels, while the DLBCL tissues are colored in with magenta pixels. The loading on PC2, Figure 1d, shows the peaks responsible for the difference between healthy and cancerous tissues. These are also listed in Table 1. The negatively loading peaks associated with cancerous samples also showed a diverse range of lipid classes. The most intensely loading peaks (PIs, PEs, and PGs) in the cancerous samples are more saturated with either 1 or 2 double bonds in total on their fatty acid chains. However, less intense loading peaks did continue to show diverse degrees of unsaturation. Further, additional lipid types were shown to coload with the cancerous signals.

**Table 1:** Most Prominent Positive and Negative Loading Peaks from PC2 of the Lymphoma Image Array

PC2 positive loading (healthy)		PC2 negative loading (cancerous)	
*m*/*z*	lipid	*m*/*z*	lipid
885.55	PI (38:4)^9,19^	1151.72	NeuAcHex_2_Cer (34:1;O2)^17^^a^
857.52	PI (36:4)^19^	1050.68	SHex_2_Cer (42:2)^20^^a^
812.55	PS (38:3)	915.59	PI (40:3)^9^
810.53	PS (38:4)	913.58	PI (40:4)^9,19^
788.54	PS (36:1)	909.55	PI (40:6)^9,19^
766.54	PE (38:4)^19^	889.58	PI (38:2)^9^
750.54	PE (O-38:5)	863.56	PI (36:1)
749.52	PA (40:5)	861.55	PI (36:2)^9^
738.51	PE (36:4)^19^	859.53	PI (36:3)^9^
722.51	PE (O-36:5)	835.54	PI (34:1)^9^
619.28	LPI (20:4)	786.53	PS (36:2)^19^
599.32	LPI (18:0)	775.55	PG (36:1)
		773.54	PG (36:2)^19^
		747.51	PG (34:1)^19^
		744.55	PE (36:1)
		742.54	PE (36:2)^19^
		740.52	PE (36:3)^19^
		716.53	PE (34:1)
		714.51	PE (34:2)^19^

a Detected as potential fragment ion. For mass accuracy measurement and metabolite identification level, see Table S1.

Specifically higher mass signals at *m*/*z* 1151.72 (NeuAcHex2Cer (34:1;O2))^17^ and *m/*z 1050.68 (SHex2Cer (42:2)).^20^ Most of the lipid assignments were based on lipids reported in DLBCL xenografts from mice^19^ and blood plasma from DLBCL-diagnosed and healthy patients.^21^

*k*-means, with *k* = 3, clusters the tissues in two clusters (plus one cluster for the non-tissue-containing pixels), cluster 3 captures samples that have been diagnosed with DLBCL, and cluster 1 captures the healthy tissue samples (Figure 1b). While the *k*-means clustering provides clearer differentiation of the tissue samples in the image, the contribution from the different ions is not as easy to assess; comparing *k*-means spectra is not as obvious as comparing positively or negatively loading peaks. This comparison of image contrast versus spectral contrast highlights the complementarity of the 2 approaches to data processing, but further complementarity can also be found if the *k*-means data are treated appropriately and compared to the PCA result.

**Figure 2:**
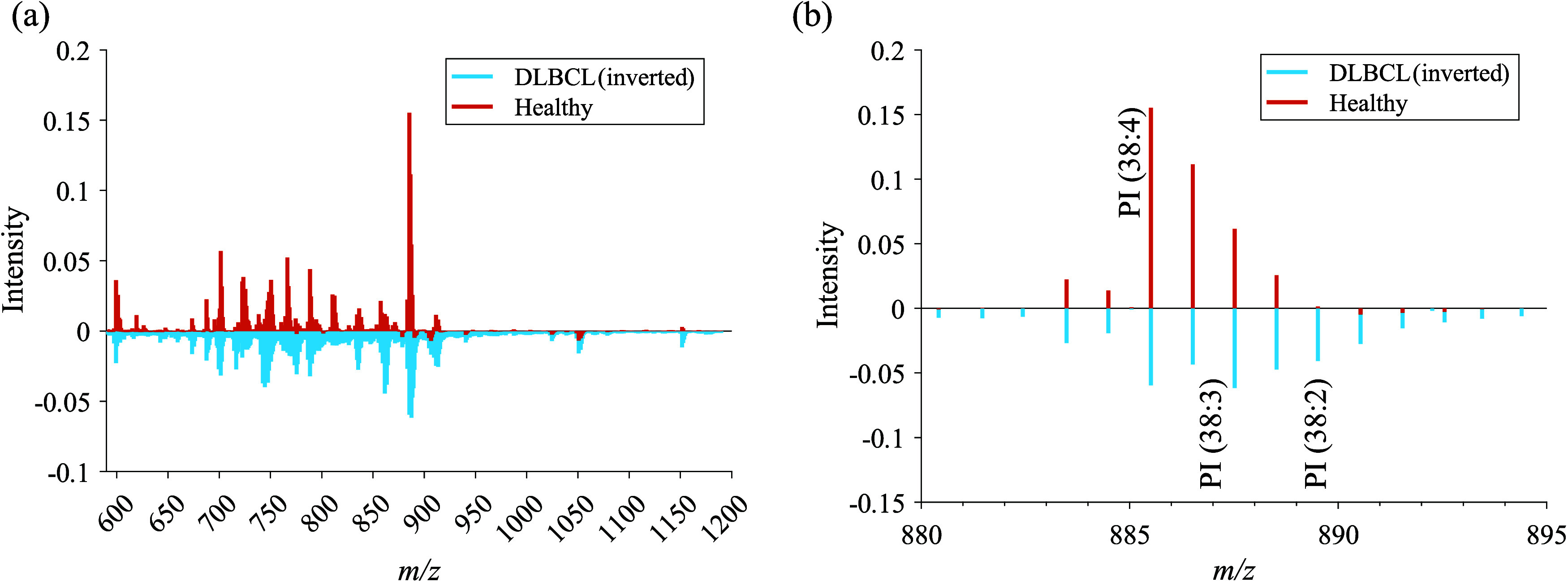
Overlayed mass spectra of DLBCL (blue, inverted) and healthy (red) from *k*-means (*k* = 3) after mean subtraction, (a) full mass range, and (b) mass range between *m*/*z* 880–890. Mass spectra from *k*-means (*k* = 3) are available in the Supporting Information.

To more clearly highlight the unique signatures of each mean spectra produced by the *k*-means analysis, the overall mean was calculated and subtracted from the cluster 1 and cluster 3 mean spectra (cancer and healthy samples respectively). Figure 2 shows these with cluster 1 in red and cluster 3 in blue, where the cluster 3 result has been inverted for clearer visualization. A close comparison of the different lipid profiles highlights differences in peak intensity between the healthy and cancerous samples that were not caught in the PCA. Inspection of the PI 38:x lipids in Figure 2b shows a higher relative intensity for PI (38:3) compared to that of PI (38:4) in the DLBCL tissues. This subtle change was confirmed in the ion images (Figure S18) as *m*/*z* 887.56 is brighter than *m*/*z* 889.58 but also localized differently to *m*/*z* 885.55 in the cancerous tissues. In PC2 (Figure 1d), PI (38:3) does not have a strong negative loading value due to isobaric interference from the M + 2 isotope of PI (38:4).

An overall comparison of the fatty acid chains of the different lipid types present in the PCA loadings discriminating between the healthy and cancerous samples indicates that PI, PS, and PE chains appear to be shorter and more saturated, while the PG lipids appear to be longer and less saturated in the cancerous samples. The lipids associated with healthy lymph tissue and DLBCL found in PC2; PI (38:4) and PI (38:2), respectively, indicate that less saturated fatty acid precursors are used for C-38 PI-lipids in the cancerous cells. Angerer et al. found that for breast cancer, PI (38:4) appears in the inflammatory tissue that surrounds the tumor while PI (38:3) and PI (38:2) were detected inside the tumor.^9^ For the samples in this study, PI (38:4) is present in the inflammatory cells that are normally found in healthy lymph nodes and lymphatic tissues. Similar to breast cancer, the cancerous cells in the lymph nodes contain the more saturated PI (38:2) and (38:3). Nilsson et al. detected PI (36:4) and PI (36:3) in basal cell carcinoma and with increased intensity in more aggressive areas of the tissues.^11^ In this lymphoma study, PI (36:4) was loaded with the healthy samples and PI (36:3) was loaded with the cancerous samples along with PI (36:2) and PI (36:1).

While the general classification of the tissue samples between normal and cancerous tissues is clear, upon close inspection specific subregions or clusters of pixels appear to be miss-assigned in some cases. This can be explained by the fact that other types of cells and tissue may have been grouped together as cancerous or noncancerous as all pixels need to be placed in the prespecified number of categories in *k*-means and all pixels will receive a score on each PC in PCA. Hence, the *k*-means is discriminating the biggest differences in tissue types and not specifically detecting cancer.

**Figure 3:**
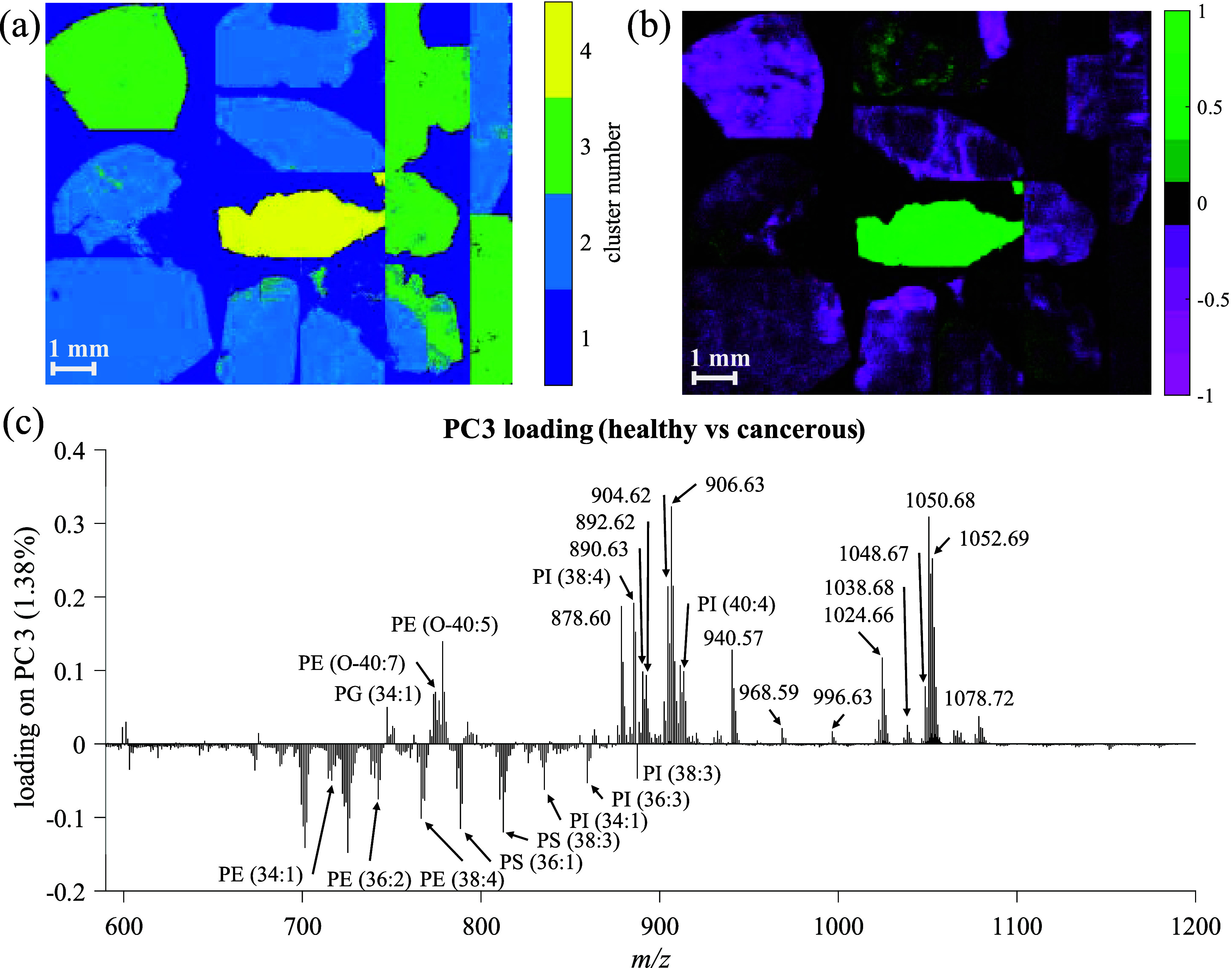
(a) *k*-means (*k* = 4) image and (b) PC3 score image of the SIMS image array and (c) annotated PC3 loading. Cluster 2 corresponds to healthy lymphoid tissues, cluster 3 to cancerous lymphoid tissues, and cluster 4 to an aggressive lymphoma subtype. Scale bar: 1 mm.

It is perhaps not unexpected that the complex tissue samples contain a wider variety of chemical compositions than simply cancer or not, and so it is useful to interrogate the data further. With PCA, this can be achieved by inspecting the scores and loadings for PCs that capture less variance than PC1 and PC2 and for *k*-means by increasing the prescribed number of clusters. Figure 3 shows the result from *k*-means clustering using four clusters and the scores image for PC3 of the PCA. The *k*-means with *k* = 4 resulted in DBLCL tissue 2 being separated as cluster 4 (yellow), Figure 3a, while PCA highlighted this in positive scoring pixels on PC3 (bright green). It is interesting to note the differences in the results from the *k*-means versus the PCA here as the *k*-means show tissue C2 to be different from the normal tissues and the other cancer tissues, while PC3 (negatively scoring pixels) shows more variation within individual tissue pieces (Figure 3b) and is picking up on different tissue types in both healthy and DBLCL tissues. The PC3 loadings are presented in Figure 3c, and the most intense peaks are listed in Table 2.

**Table 2:** Most Prominent Positive and Negative Loading Peaks from PC3 of the Lymphoma Image Array

PC3 positive loading (highly aggressive)		PC3 negative loading (other tissues)	
*m*/*z*	lipid	*m*/*z*	lipid
1078.72	SHex_2_Cer (44:2)^20^^a^	887.56	PI (38:3)
1052.69	SHex_2_Cer (42:1)^20^^a^	859.53	PI (36:3)
1050.68	SHex_2_Cer (42:2)^20^^a^	835.54	PI (34:1)
1048.67	SHex_2_Cer (42:3)^20^^a^	812.55	PS (38:3)
1024.66	SHex_2_Cer (40:1)^20^^a^	788.54	PS (36:1)
996.63	SHex_2_Cer (38:1)^20^^a^	766.54	PE (38:4)
968.59	SHex_2_Cer (36:1)^20^^a^	742.54	PE (36:2)
940.57	SHex_2_Cer (34:1)^20^^a^	716.54	PE (34:1)
913.58	PI (40:4)		
906.63	SHex_2_Cer (42:1) (OH)^20^^a^		
904.62	SHexCer (42:2) (OH)^20^^a^		
892.62	SHexCer (41:1) (OH)^20^^a^		
890.63	SHexCer (42:1)^20^^a^		
885.55	PI (38:4)		
878.60	SHexCer (40:1) (OH)^20^^a^		
778.57	PE (O-40:5)		
774.5*5*	PE (O-40:7)		
747.51	PG (34:1)		

a Identified as a possible fragment ion. For mass accuracy measurement and metabolite identification level, see Table S1.

The positive loadings for PC3 show that DLBCL tissue 2 correlates mainly with various hexosyl ceramides (HexCer) and sulfatides (SHexCer) along with some phosphatidylglycerol species. NeuAcHex_2_Cer (34:1;O2) could potentially be a fragment from a larger GM3 ganglioside,^22^ while SHexNacHex_2_Cer (42:2) may be a fragment of a larger sulfoglycosphingolipid^20^ as despite the softer ionization afforded by the use of GCIBs, in-source fragmentation is still common with SIMS. Jirásko et al. analyzed SHexCer lipids in renal cell carcinoma tissues and presented extensive fragmentation pathways using MALDI-orbitrap and MS/MS.

Comparing the pathways with the fragments produced by GCIB, the positive loading ions shown in Figure 3c and Table 2 are suggested to be fragmented SHexCer lipids such as SHexNacHex_2_Cer (44:2), detected with loss of the N-acetylhexoseamine-H_2_O group at *m*/*z* 1078.72 as [M – C_8_H_13_NO_5_]^−^ but identified as SHex_2_Cer (44:2), and SHex_2_Cer (42:1) (OH), detected with the loss of a monosaccharide at *m*/*z* 906.63 as [M – C_6_H_10_O_5_]^−^ and identified as SHexCer (43:1).^20^ The negative PC3 loadings are dominated by a range of PS and PE lipids along with PI (34:1), PI (36:3), and PI (38:3).

The initial patient diagnosis based on the DLBCL tissue 2 biopsy involved various antibody staining’s for signature proteins. Inspection of this archived data provided an explanation of the different characters of this sample. The K_i_-67 staining, an indicator of cellular proliferation, scored >90% in this sample indicating highly proliferating cancerous cells from a very aggressive subtype of DLBCL, see Figure S19 for K_i_-67 antibody-stained images of DLBCL tissue 1 compared to tissue 2. For DLBCL, a typical K_i_-67 index value is around 70–80%, varying by subtypes.^23^

**Figure 4:**
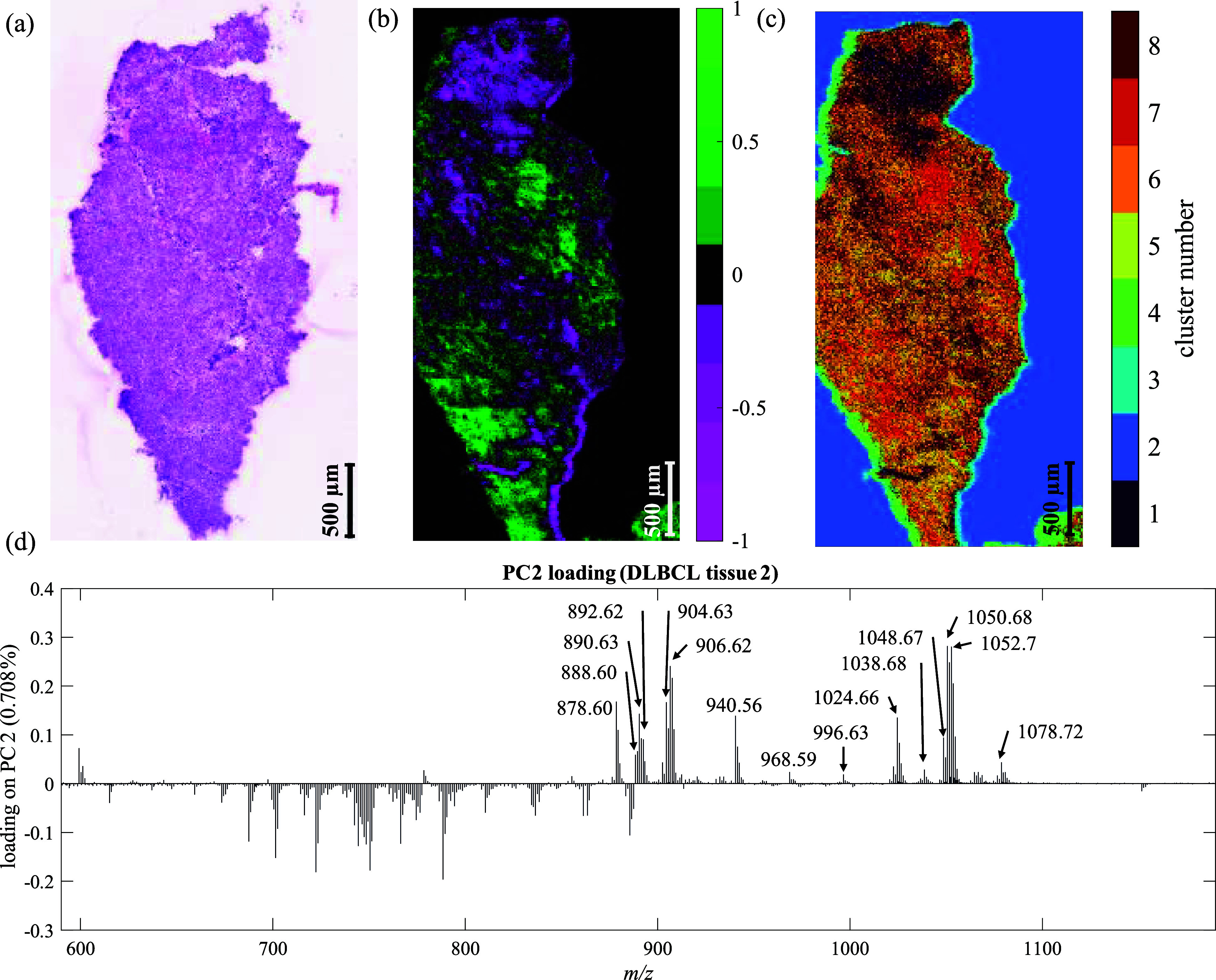
Images and loadings for DLBCL tissue 2. (a) H&E image, (b) *k*-means (*k* = 8) image, (c) PCA score image from PC2, and (d) annotated PC2 loading. Scale bar: 500 μm. Note that the tissue image is rotated 90° compared to the composite tissue images shown in Figure 1. Additional PCA and *k*-means results are available in the Supporting Information.

The GCIB-SIMS data clearly contained sufficient information to identify cancer and also variation in cancer types. To investigate the level of fine detail that could be extracted from the images, the highly aggressive sample was subjected to further MVA as an individual tissue image. *k*-means was performed multiple times using 3–10 clusters, Figure S21 shows the result for *k*-means analysis using *k* = 3–10, while the result for *k* = 8 that appeared to capture the most regions of the tissue without becoming excessively noisy is shown in Figure 4c along with the H&E-stained image of a consecutive section and the scores plot for PC2 from the PCA analysis of this section.

In the PC2 scores image, the areas with positive scoring pixels coloaded with peaks detected between *m*/*z* 850–1100, *k*-means (*k* = 8) separated parts of these areas into cluster 7 as shown in Figure 4. Pathological assessment of these areas deemed that they contained increased amounts of apoptotic cells. Sulfatides have been associated with increased cell apoptosis in cancer cells,^24^ suggesting that the apoptotic cells are the source of the assigned sulfatides.

### Other Anomalies during Cancer Classification

**Figure 5:**
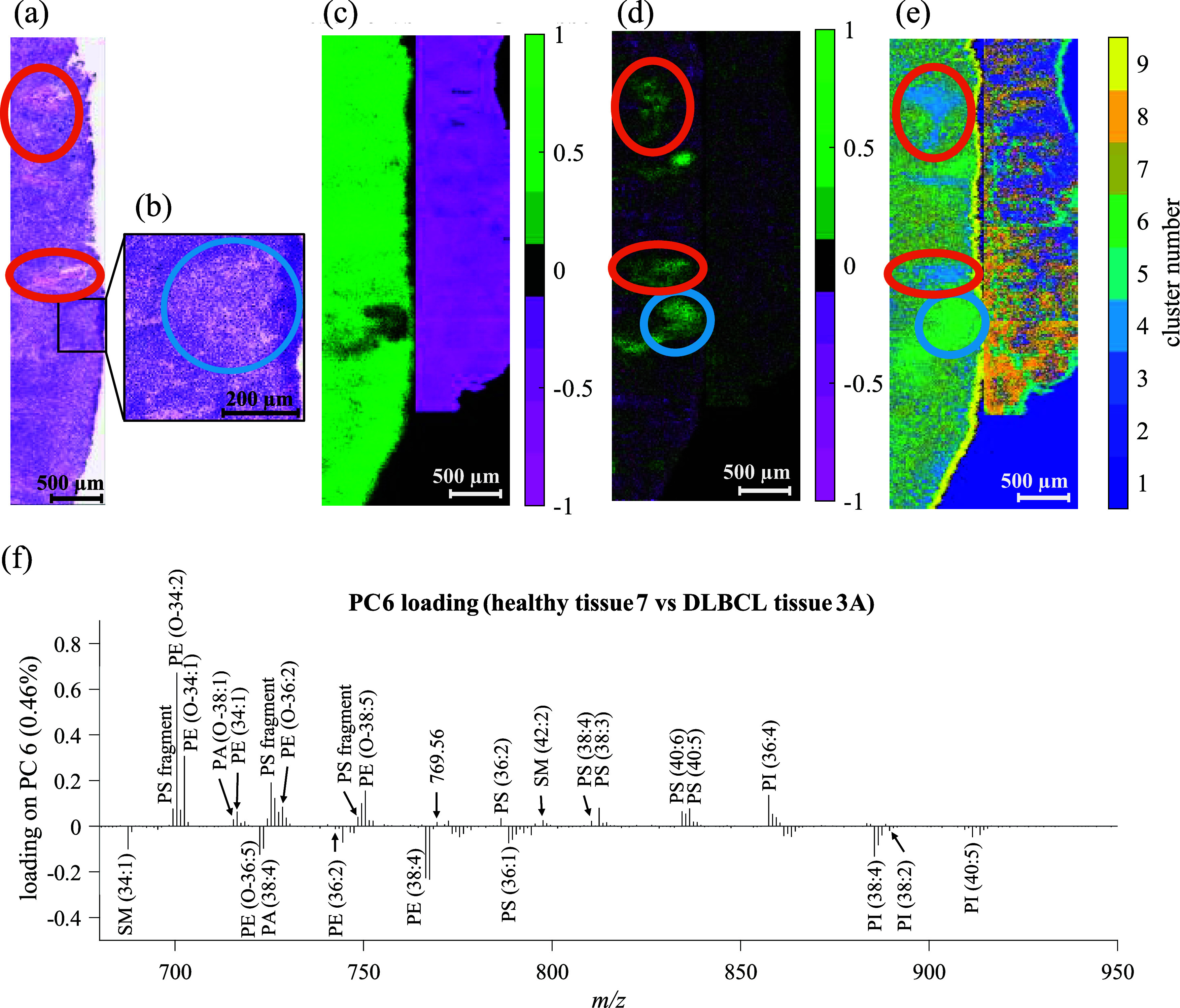
(a) H&E image of healthy tissue H7 (connective tissue marked with orange ring) and (b) zoomed-in image on histiocytes (blue ring). Images and loadings from combined SIMS images of healthy tissue H7 and DLBCL tissue C3A. (c) PC2 scores image of H7 (positive score, green) and C3A (negative score, magenta). (d) PC6 scores image with histiocytes (blue ring) and connective tissue (orange ring) highlighted assigned positive score (green). (e) *k*-means (*k* = 9) image, connective tissue assigned to cluster 4, and histiocytes assigned to cluster 6. (f) Annotated PC6 loading at *m*/*z* 680–950. Scale bar: 500 μm for (a) and (c–e), and 200 μm for (b). The full mass range for PC6 loading, additional PCA, and *k*-means results are available in the Supporting Information.

In tissue H7, presumably healthy tissue, an area was clustered with cancerous samples by PCA and *k*-means (Figure 1a,b). This area in the tissue was found in the H&E image with increased presence of histiocytes, see Figure 5a. This sample comes from a patient that has previously been treated for Hodgkin’s lymphoma; the treatment has probably caused changes in the tissue resulting in among other increased appearance of histiocytes which in turn is picked up by PCA and *k*-means. Figure 5a,b shows the histiocytes with optical resolution that can discern individual cells. The SIMS images were acquired with 7 μm pixel size; this is too low to distinguish the signal from these individual cells, and the lipid composition is associated with the histiocyte-rich region as a whole.

To further investigate the difference between the histiocytes and lymphocytes, PCA was used on tissue H7 together with tissue C3A. This combination provided within image reference areas healthy and cancerous tissue. The scores image for PC2, see Figure 5c, shows the histiocytes having a low score that varies between positive and negative. The histiocytes have the best separation on PC6 and received a positive score but together with connective tissue in tissue H7 as seen in Figure 5d.

The loading for PC6, shown in Figure 5f, was used to collect ion images from 21 positive loading peaks (*m*/*z* 599.32, 699.50, 700.53, 702.53, 715.57, 716.54, 718.55, 725.52, 728.55, 748.52, 749.52, 750.54, 769.56, 772.57, 786.53, 797.64, 810.53, 812.55, 834.53, 836.55, and 857.52) and 21 negative loading peaks (*m*/*z* 687.55, 722.52, 723.50, 742.54, 744.55, 746.52, 747.51, 766.54, 767.55, 773.54, 775.55, 778.57, 788.54, 792.55, 794.56, 861.55, 863.57, 885.55, 889.58, 911.57, and 913.58), see Figure S26, to compare the relative intensity of lipids between the histiocytes and the other regions from H7. From inspection of the ion images, the histiocytes can be differentiated based on the 26 peaks shown in Figure 5f and detailed in Table 3.

**Table 3:** Positive and Negative Loadings of Lipids with Relative Signal to Histiocytes, and between Healthy Tissue 7 and DLBCL Tissue 3A^a^

*m*/*z*	loading	lipid	localized to histiocytes
911.57	negative	PI (40:5)	·↑
889.58	negative	PI (38:2)	·↑
885.55	negative	PI (38:4)	↓↓
857.52	positive	PI (36:4)	↑↑
836.55	positive	PS (40:5)	·↑
834.53	positive	PS (40:6)	↑↑
812.54	positive	PS (38:3)	·↑
810.53	positive	PS (38:4)	↓
797.64	positive	SM (42:2)^17^	·↑
788.54	negative	PS (36:1)	↓↓
786.53	positive	PS (36:2)	·↑
769.56	positive		·↑
766.54	negative	PE (38:4)	↓
750.54	positive	PE (O-38:5)	↓
749.52	positive	PS (40:5)^b^	·↑
742.54	negative	PE (36:2)	·↑
728.55	positive	PE (O-36:2)	↑
725.52	positive	PS (38:3)^b^	·↑
723.50	negative	PA (38:4)	↓
722.51	negative	PE (O-36:5)	↓↓
716.54	positive	PE (34:1)	↑
715.57	positive	PA (O-38:1)	↑
702.53	positive	PE (O-34:1)	↓
700.53	positive	PE (O-34:2)	↑↑
699.50	positive	PS (36:2)^b^	·↑
687.55	negative	SM (34:1)^17^	·↓

a Increase means higher intensity and decrease means lower intensity in the histiocytes compared to the remaining cells in healthy tissue H7. ·↑/·↓: slight difference. ↑/↓: small difference. ↑↑/↓↓: moderate difference. For mass accuracy measurement and metabolite identification level, see Table S1.

b PS fragment ion, [M – H – C_3_H_5_O_2_N]^−^.

While the peaks were either correlated or anticorrelated with the histiocyte region, no peak was specific to these cells, and in fact, many ions were actually most intense in either the cancerous or healthy parts of the tissue samples as shown in the individual ion images (Figure S26). While PCA seemed to group the histiocyte region with other areas of connective tissue, *k*-means (*k* = 9) grouped the connective tissue into cluster 4 and the histiocytes into cluster 6 as seen in Figure 5e. This further supports the benefits of using *k*-means for imaging analysis of intact lipids and highlights the ability to identify various types of cells with low variance in lipid composition. Hence, multivariate methods are needed to identify multiple subtle changes in the lipidome associated with different cell types. However, “go to” techniques such as PCA struggle to highlight small regions of unique chemistry in large data sets, as the contribution of these relatively few pixels is small compared to the overall variance in the data set.

## Conclusions

The use of the GCIB provides sufficient sensitivity to allow intact lipid imaging of human lymphoid tissue. In the first instance, the information-rich data can be used to elucidate chemical differences between malignant and nonmalignant samples and beyond this also identify different types of lymphoma samples (more or less aggressive) and also highlight small subpopulations of cells representing histiocytes. Combined with MVA, GCIB-SIMS has potential in the future to be included in the pathological assessment of tissues as a diagnostic tool.

While other types of cancer often lead to an increase in inflammatory cells and their lipids, lymphoma shows the opposite trend, where the onset of cancer reduces the typical inflammatory characteristics of the tissue.

Many historic MS studies have not been performed with sufficient spatial resolution to resolve tumor cells from inflammatory cells, and the inflammatory-related signal is often associated with cancer. In lymphoma, the malignant transformation reduces the proportion of healthy inflammatory cells in the MS image, and so some of these inflammatory-associated lipids, notably PI (38:4), are anticorrelated with lymphoma.

The study highlights the complementarity between image clustering techniques such as *k*-means and also the challenges associated with interpretation of the data, even following dimensionality reduction by MVA.

The results also highlight the complexity of studying such systems due to the heterogeneity of the samples, even within this relatively small cohort. Combining multiple images into a single data set facilitates the detection of global trends, e.g., cancer/healthy, but, when using current common clustering approaches PCA/*k*-means, makes characterization of smaller features in the large data set difficult. This could be counteracted by performing additional data processing on subsets of the data but will represent a greater challenge on larger cohort data sets in the future. Emerging machine learning approaches (e.g., autoencoders, or self-organizing maps) may provide potential solutions or at least advantages.^25^ Future improvements in the detail of the SIMS images, specifically moving toward cellular precision, are expected through the introduction of higher-resolution ion beams and increased instrument sensitivity to exploit them along with the incorporation dual-polarity analysis.

## Supporting Information Available

MATLAB code for peak picking, additional H&E stain images, K_i_-67 immunology images, PCA scores images and loadings, *k*-means images and mass spectra, single-ion images, an extended peak identification table including mass accuracy and metabolite identification level, and a LIPID MAPS suggested identification table (PDF)
