# Supplementary material for: Mapping the Molecular Landscape of Human DLBCL by GCIB-SIMS
Source: Anal Chem. 2025 Mar 26;97(13):7186–94. doi: 10.1021/acs.analchem.4c06594 (PMC11983375; doi:10.1021/acs.analchem.4c06594)
Supplement: Supplementary file 1 — ac4c06594_si_001.pdf [file ac4c06594_si_001.pdf]

Supporting information

Mapping the molecular landscape of human DLBCL by GCIB-SIMS

Simon Uzoni[1], Daniele Zanchin[1], Vasilis Chatzikyriakos[2,3], Noora Neittaanmäki[2,3], John S. Fletcher[1]*

1. Department of Chemical and Molecular Biology, University of Gothenburg, Gothenburg Sweden

2. Department of Laboratory Medicine, Institute of Biomedicine, Sahlgrenska Academy, University of Gothenburg, Gothenburg, Sweden

3. Region Västra Götaland, Sahlgrenska University Hospital, Department of Clinical Pathology and Cytology, Gothenburg, Sweden

*Corresponding author: john.fletcher@chem.gu.se

Supplemental method 1: Matlab code.

```
file = uigetfile('*.xlsx');
[num,txt,raw] = xlsread(file);

oldmass = num(2:end,1);
data = num(2:end, 2:end);

x = ChiMSSpectralCollection(oldmass, data');

%Select each spectra sections range.
range1 = x.keeprange(590,800);
range2 = x.keeprange(800,1199);

%Peakpicking each region. numberlimit is the n most intense peaks that will be
peak picked. d2windows
Range1picked = range1.peakdetect('numberlimit',1000,'d2window',15,'nofig');
Range2picked = range2.peakdetect('numberlimit',1000,'d2window',25,'nofig');
```

Supplemental figures:

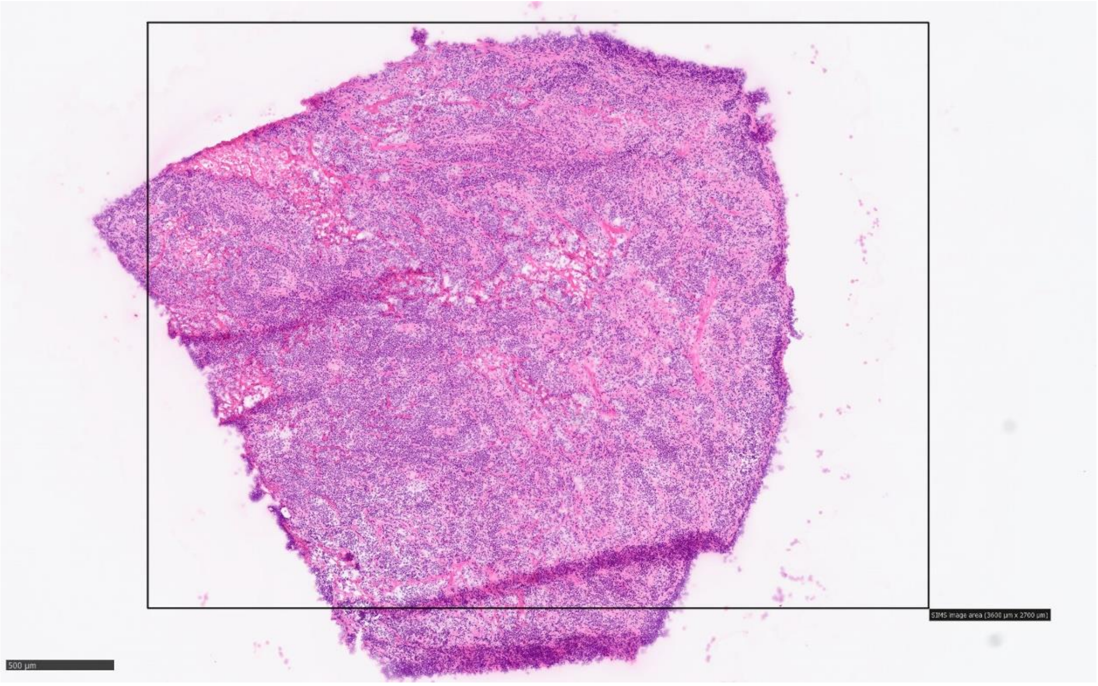

Figure S1: H&E image of DLBCL tissue 1. Scale bar: 500 µm. Black rectangle: approximated SIMS analysis area.

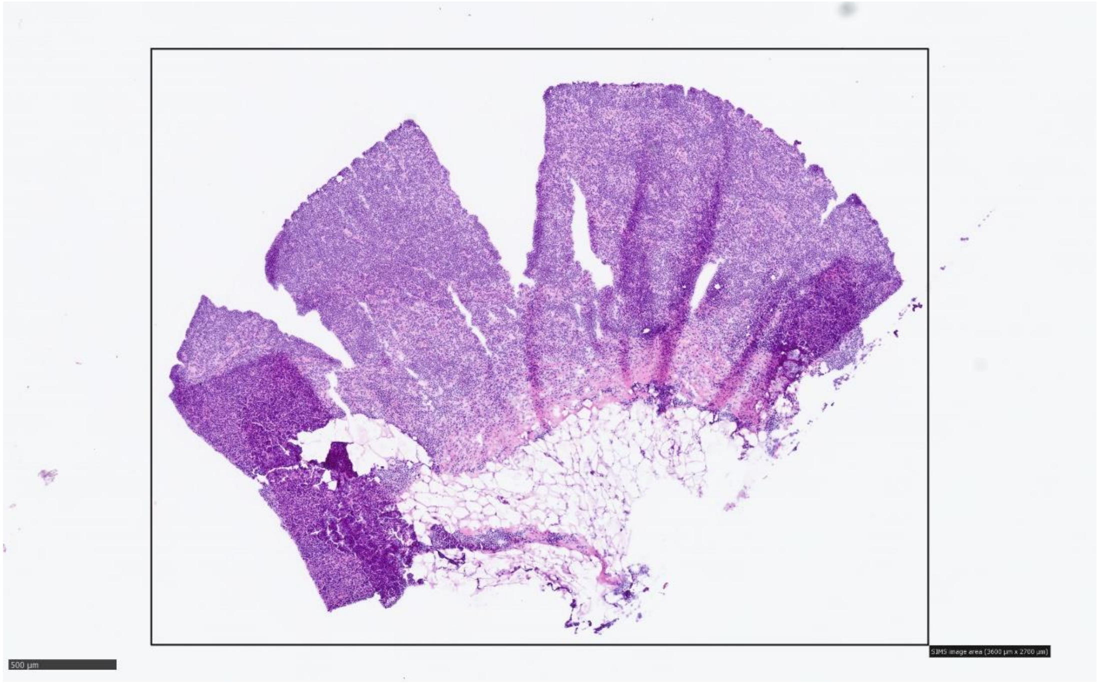

Figure S2: H&E image of DLBCL tissue 2. Scale bar: 500 µm. Black rectangle: approximated SIMS analysis area.

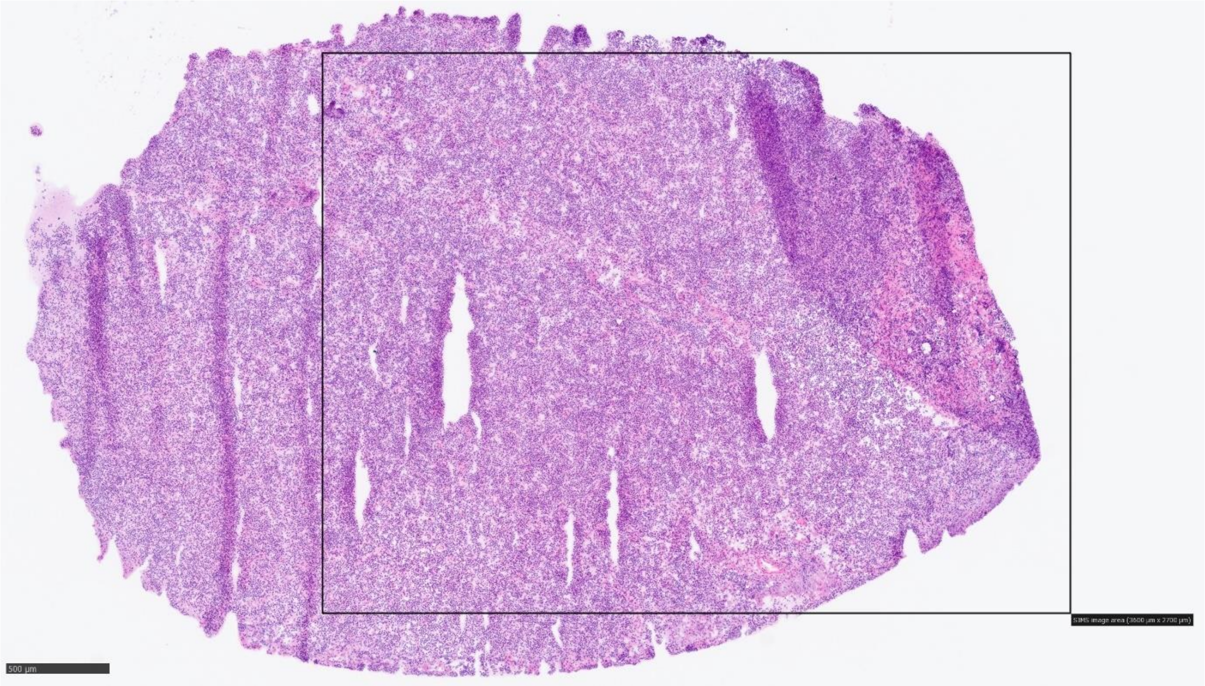

Figure S3: H&E image of Healthy tissue 2. Scale bar: 500 µm. Black rectangle: approximated SIMS analysis area.

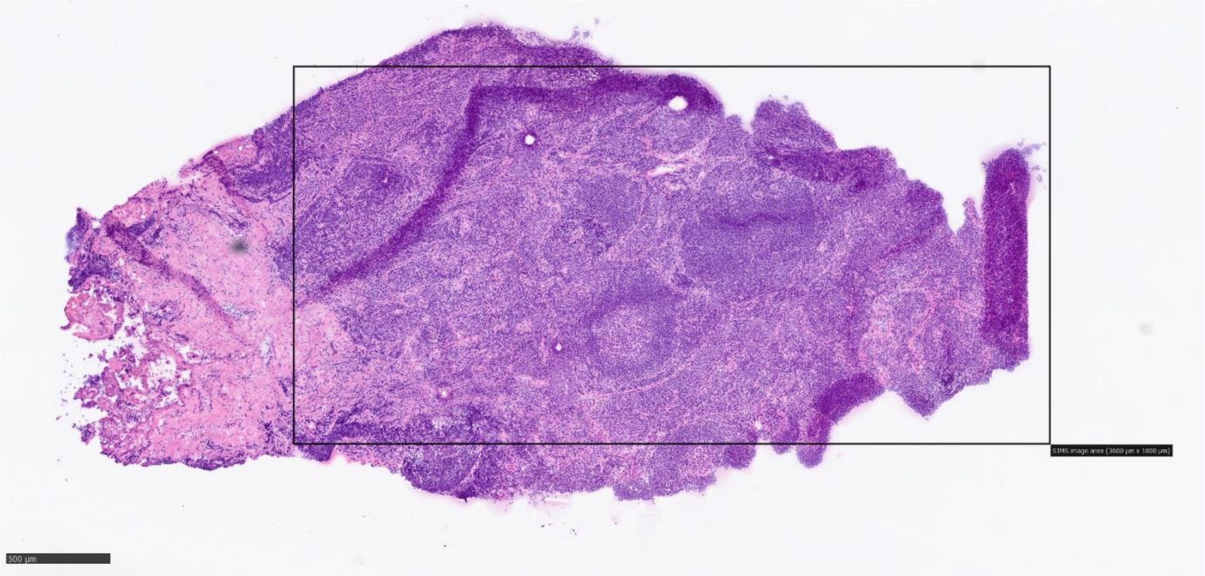

Figure S4: H&E image of Healthy tissue 3. Scale bar: 500 μm. Black rectangle: approximated SIMS analysis area.

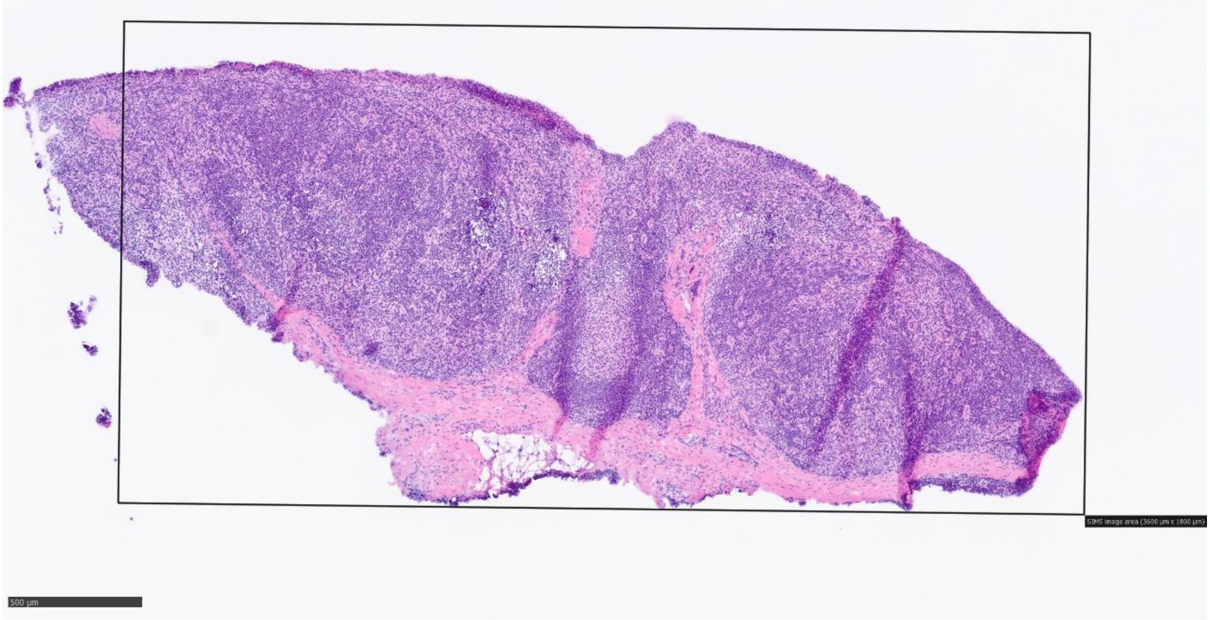

Figure S5: H&E image of Healthy tissue 4. Scale bar: 500 μm. Black rectangle: approximated SIMS analysis area.

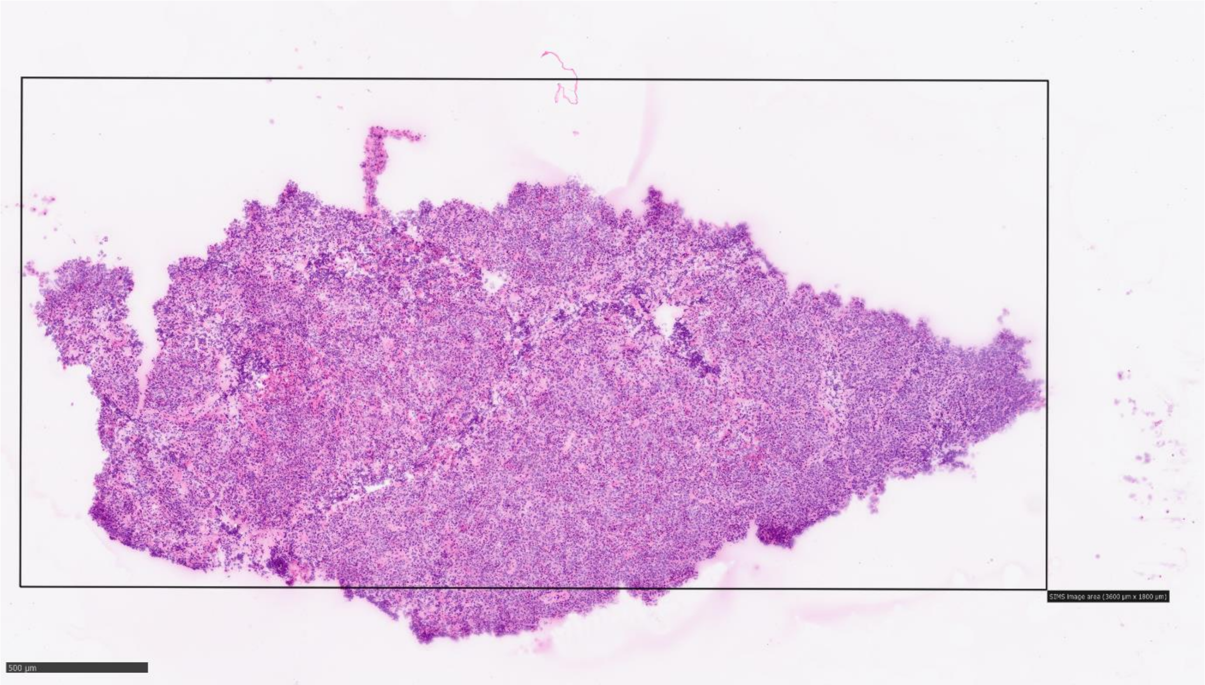

Figure S6: H&E image of DLBCL tissue 2. Scale bar: 500 µm. Black rectangle: approximated SIMS analysis area.

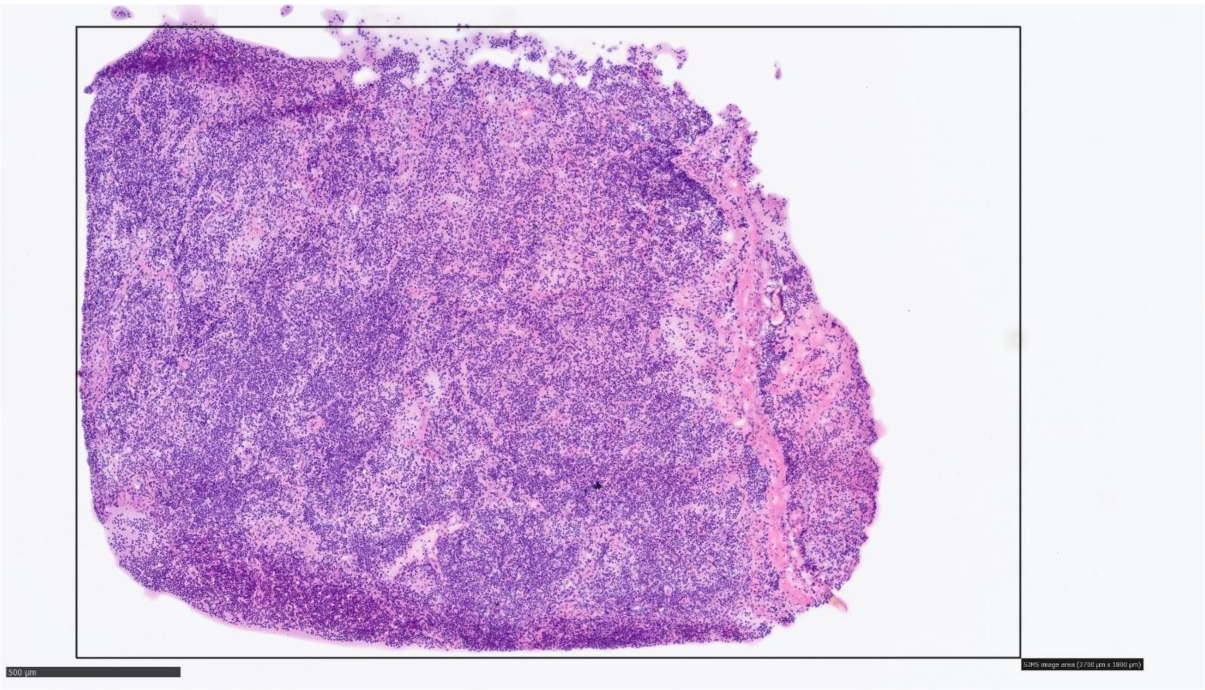

Figure S7: H&E image of Healthy tissue 5. Scale bar: 500 µm. Black rectangle: approximated SIMS analysis area.

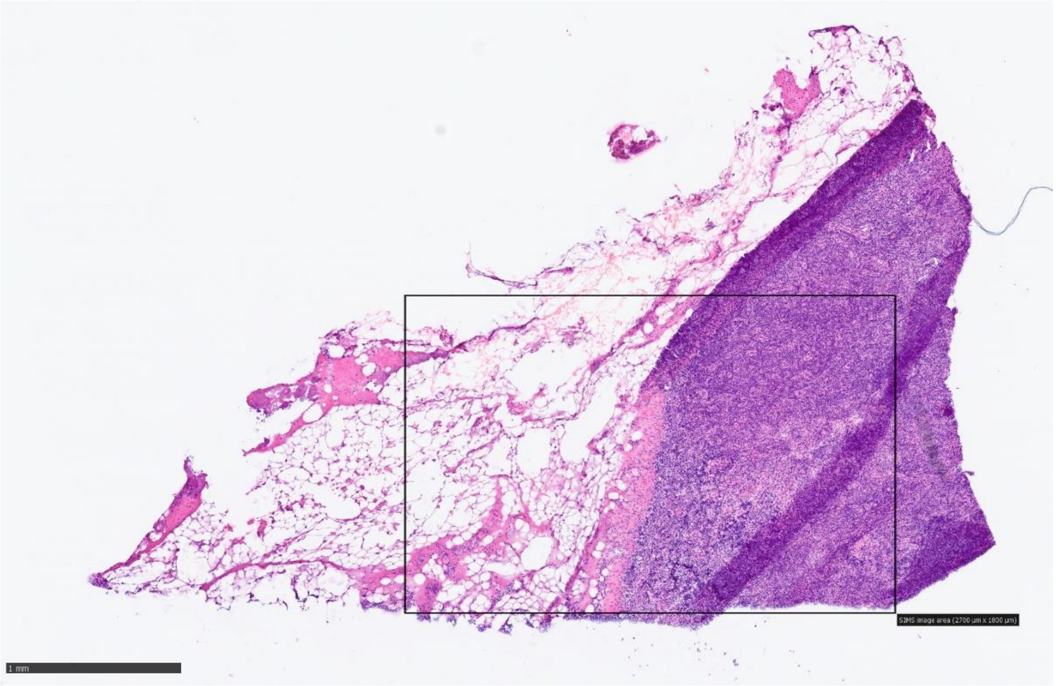

Figure S8: H&E image of Healthy tissue 6. Scale bar: 500 µm. Black rectangle: approximated SIMS analysis area.

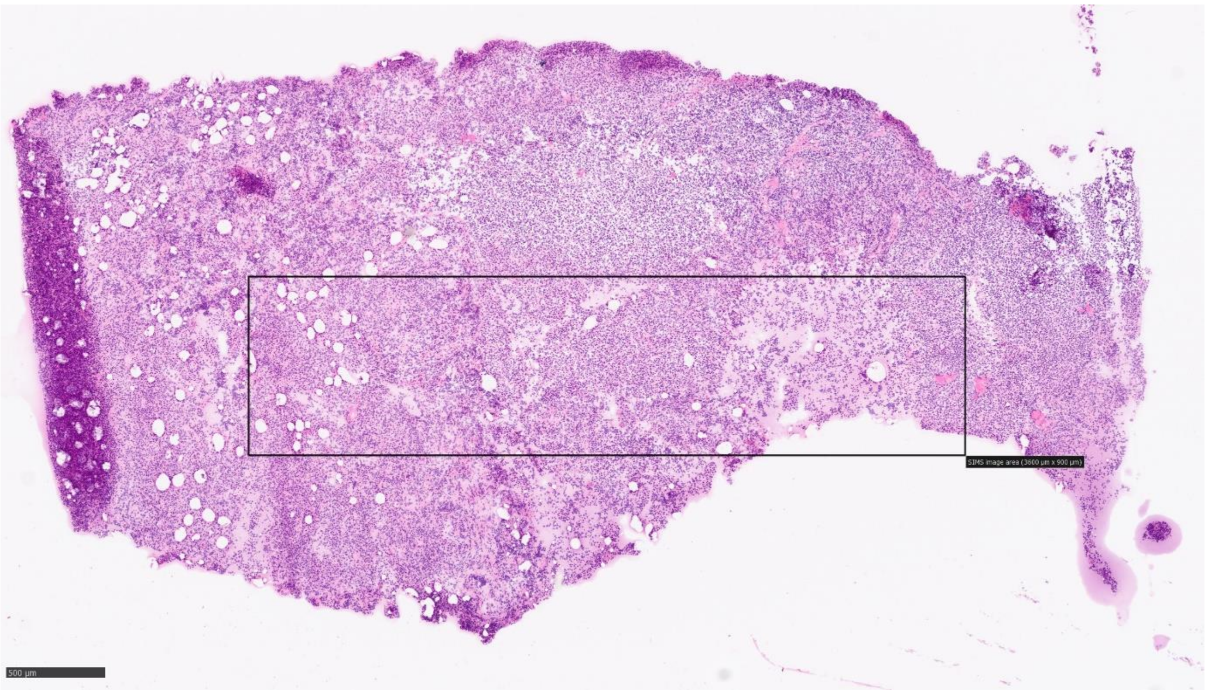

Figure S9: H&E image of DLBCL tissue 3A. Scale bar: 500 µm. Black rectangle: approximated SIMS analysis area.

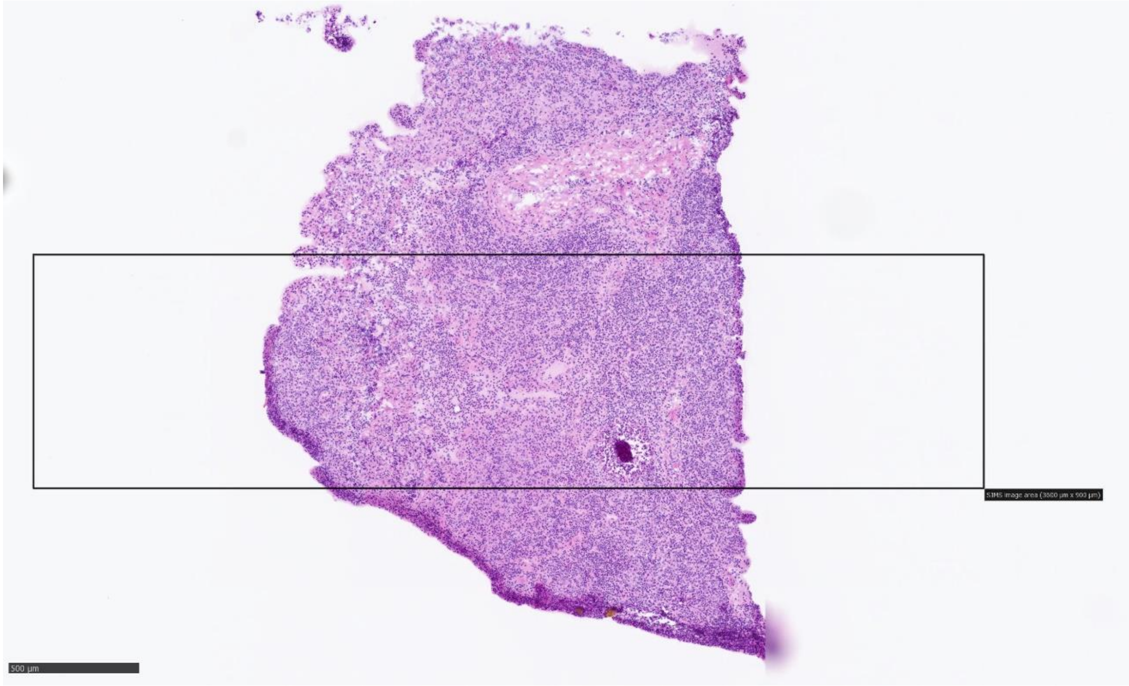

Figure S10: H&E image of DLBCL tissue 4A. Scale bar: 500 µm. Black rectangle: approximated SIMS analysis area.

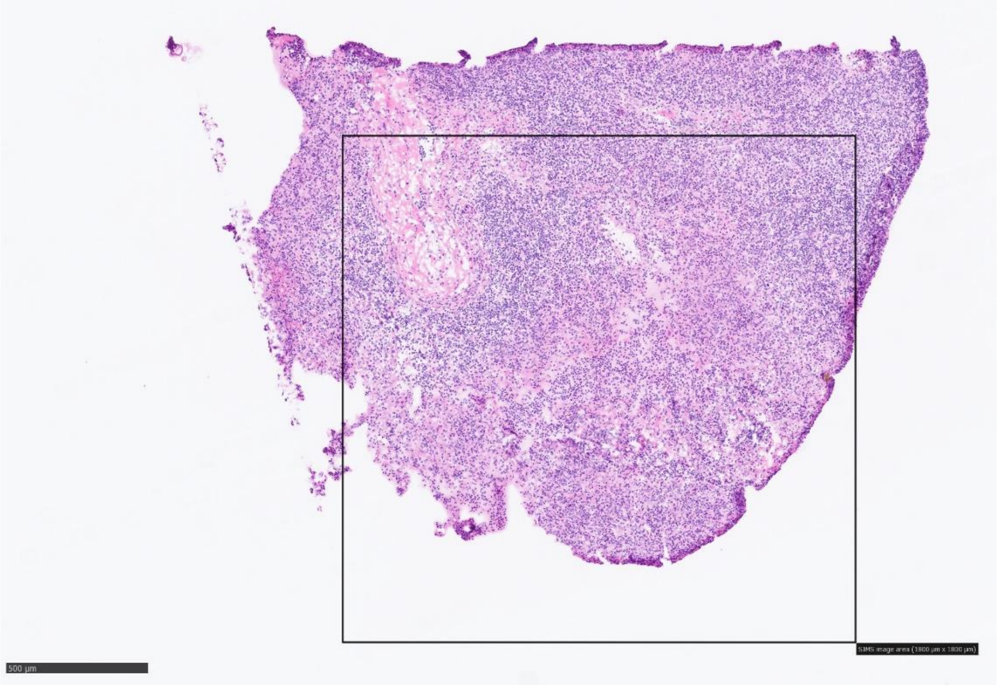

Figure S11: DLBCL tissue 4B. Scale bar: 500 µm. Black rectangle: approximated SIMS analysis area.

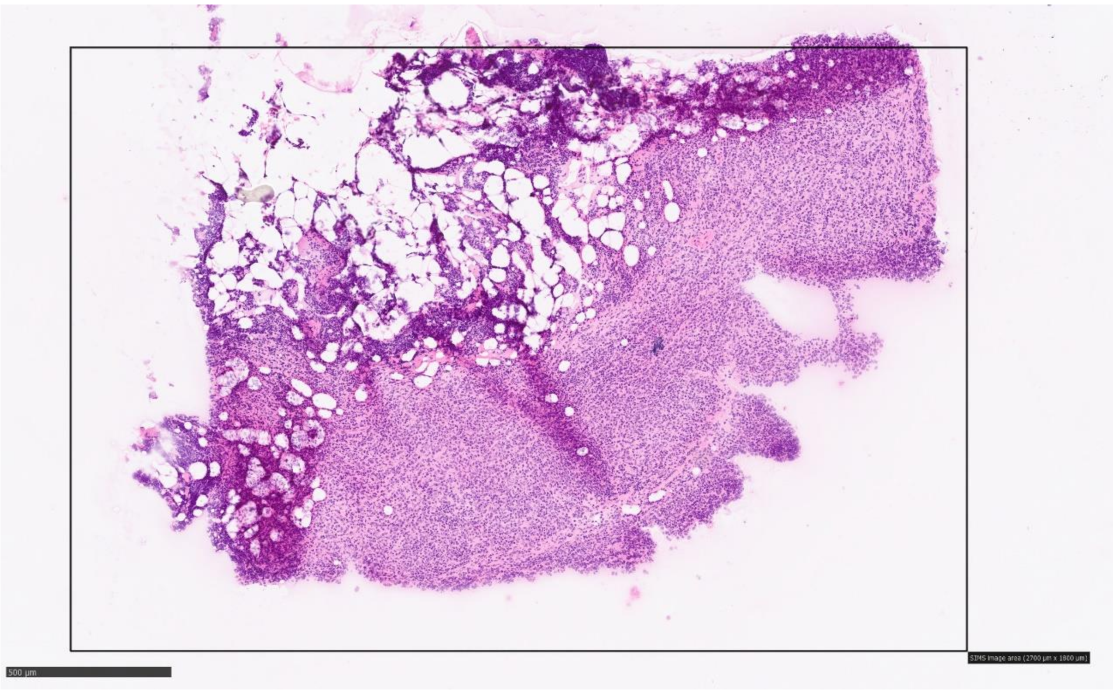

Figure S12: DLBCL tissue 5. Scale bar: 500 µm. Black rectangle: approximated SIMS analysis area.

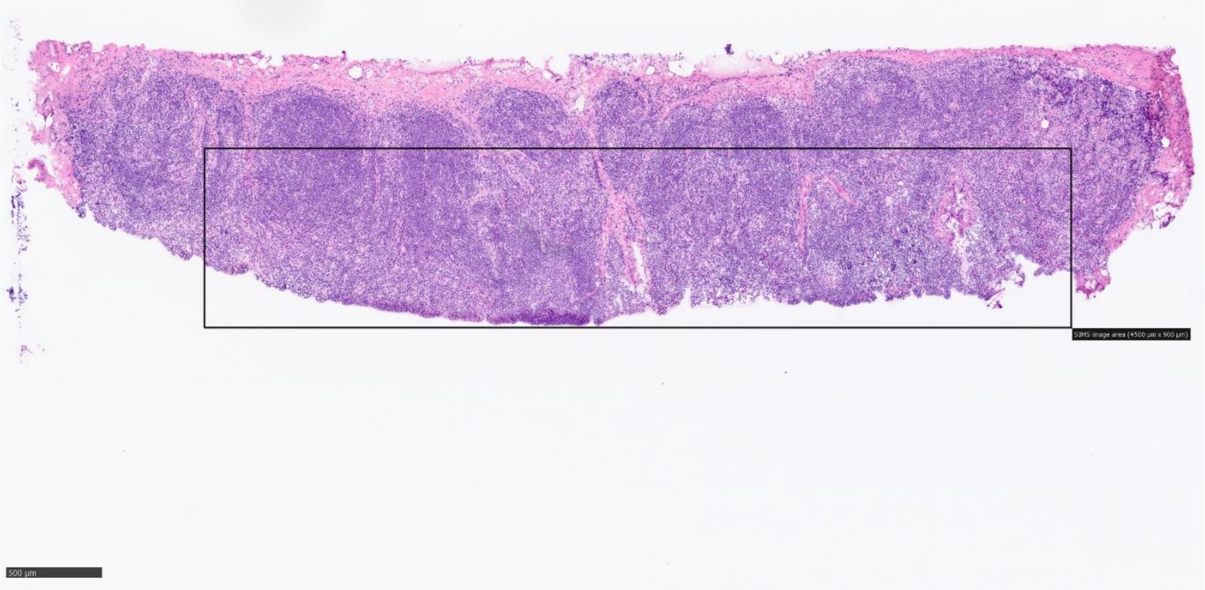

Figure S13: Healthy tissue 7. Scale bar: 500 µm. Black rectangle: approximated SIMS analysis area.

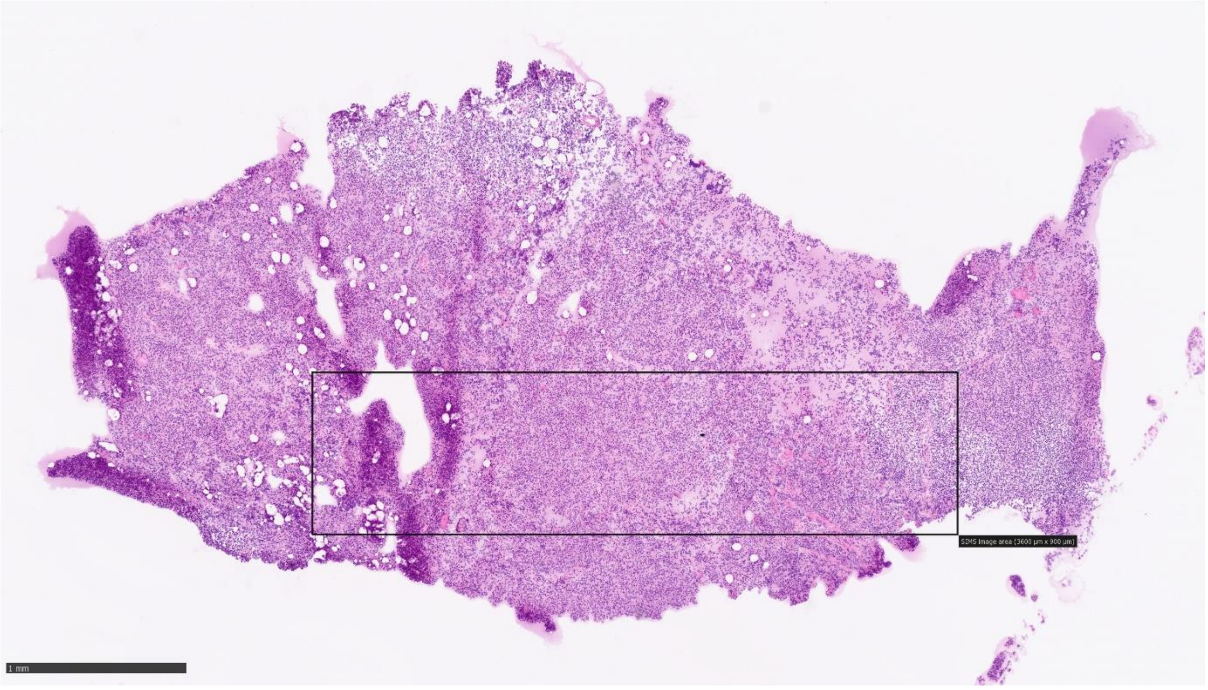

Figure S14: DLBCL tissue 3B. Scale bar: 500 µm. Black rectangle: approximated SIMS analysis area.

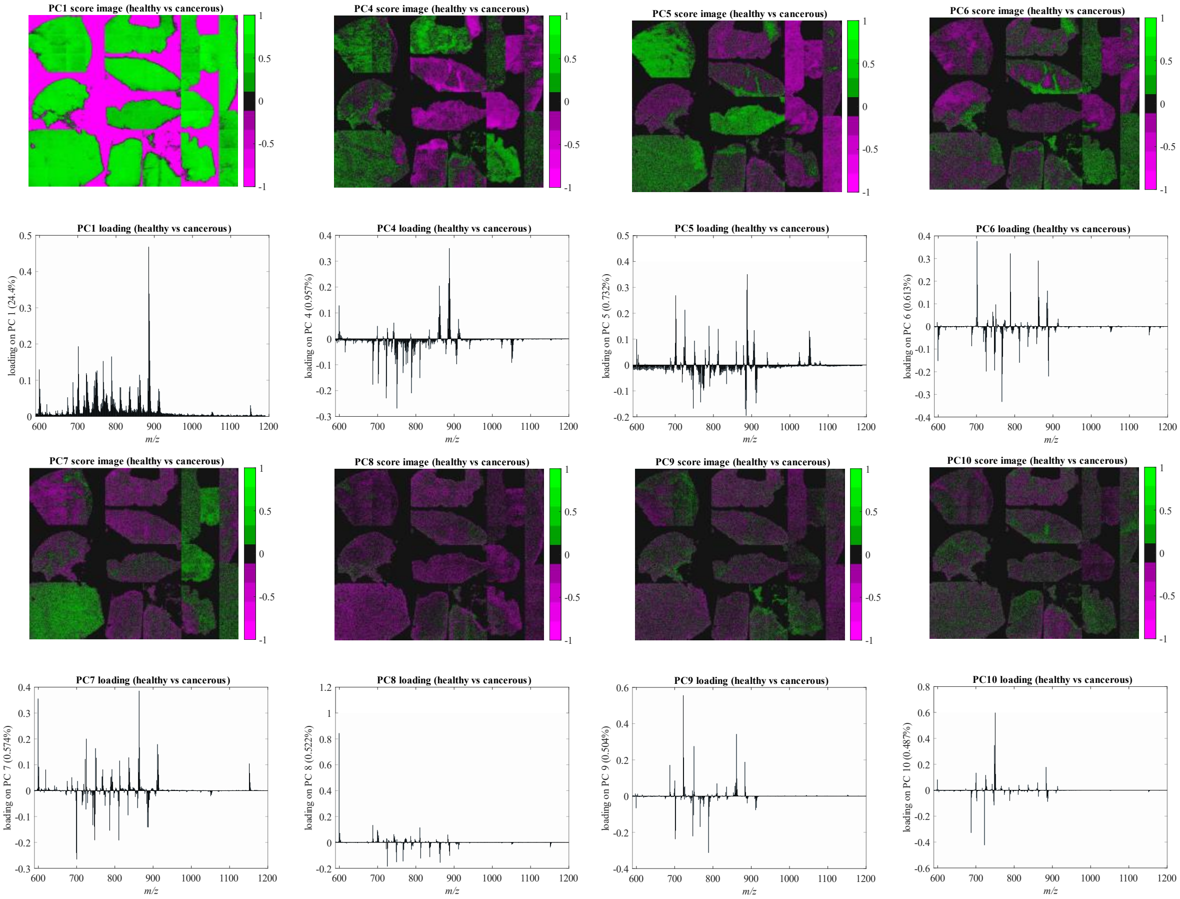

Figure S15: PCA results for lymphoid tissue array for PC 1 and 4-10.

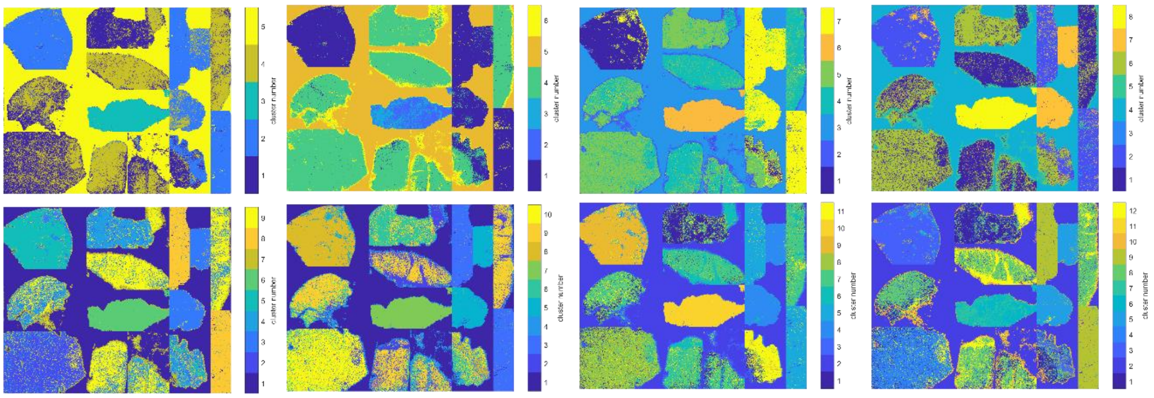

Figure S16: K-means images (k = 5-12) for lymphoid tissue array.

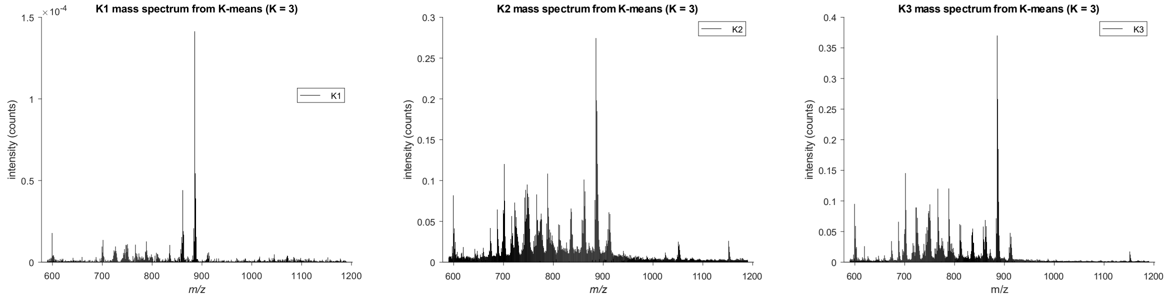

Figure S17: Mass spectra from k-means (k = 3) for lymphoid tissue array.

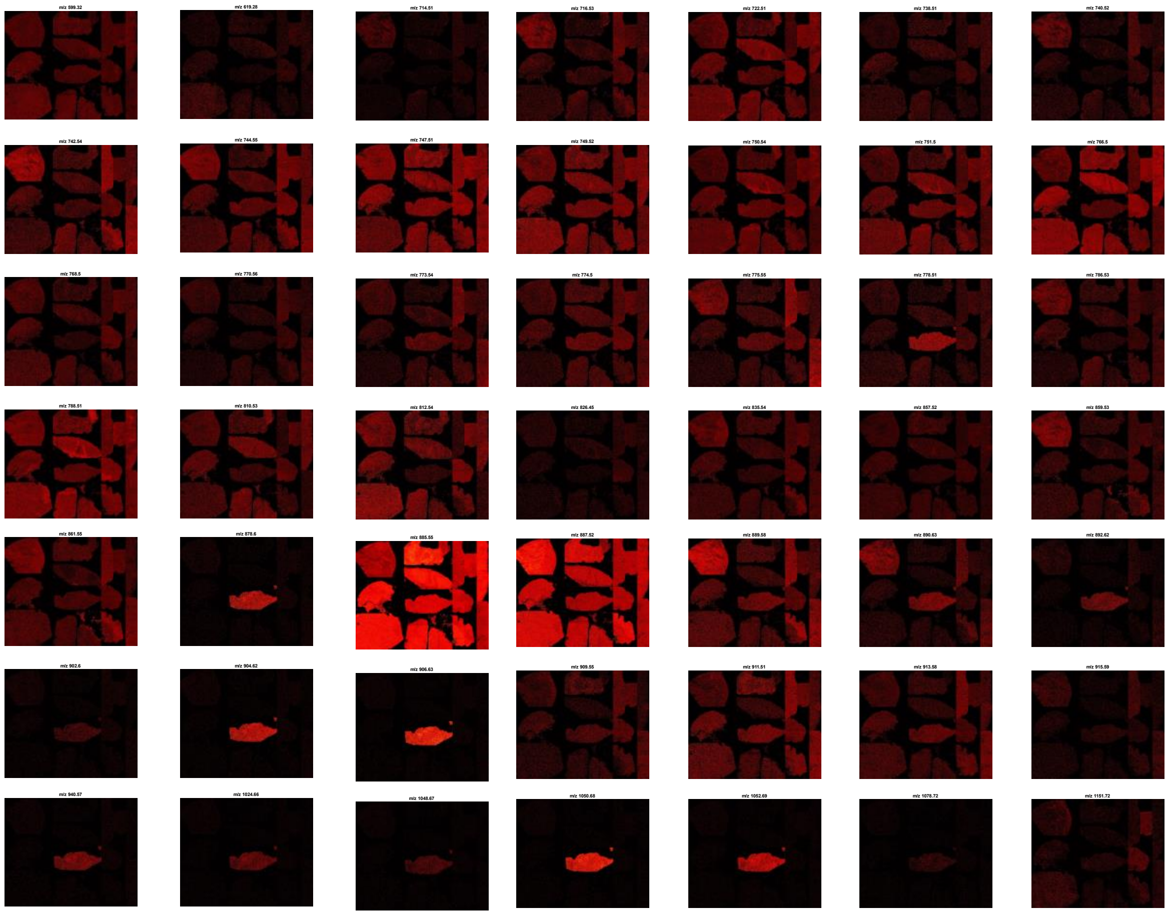

Figure S18: Ion images for lymphoid image array.

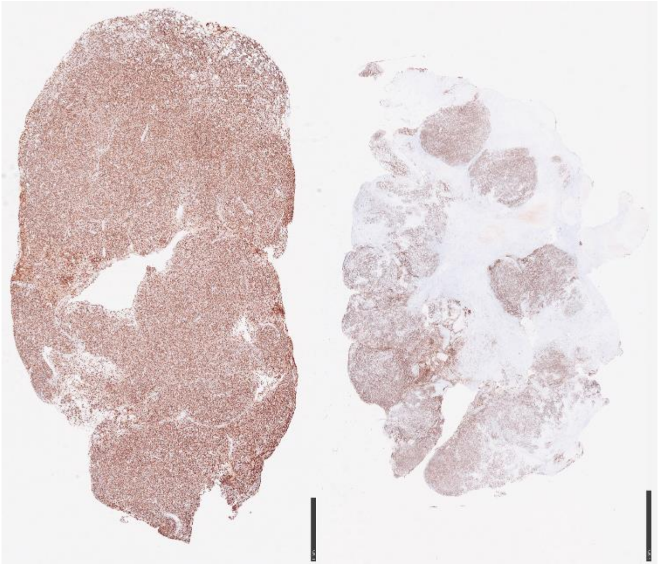

Figure S19: Ki-67 antibody staining images of DLBCL tissue 2 (left, 1mm scale bar) and DLBCL tissue 1 (right, 5mm scale bar).

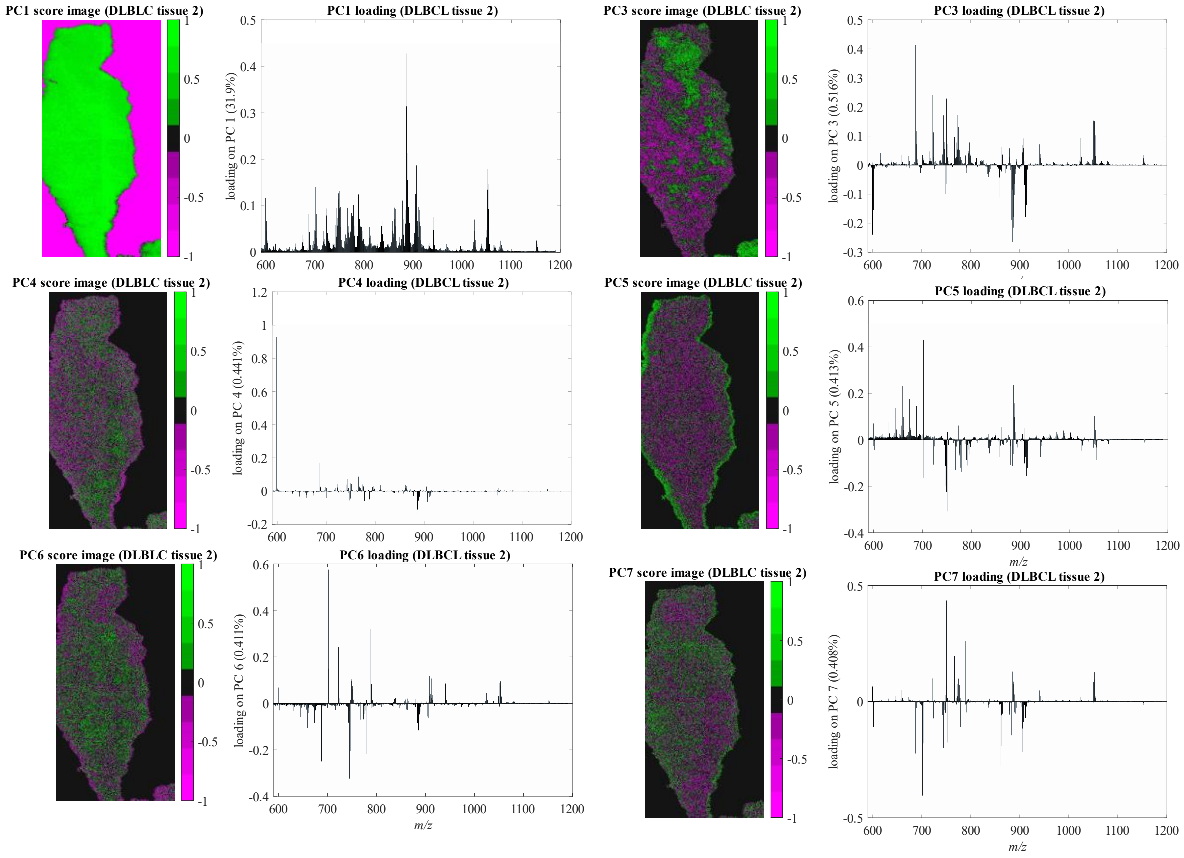

Figure S20: PCA results for DLBCL tissue 2 for PC 1 and 3-7.

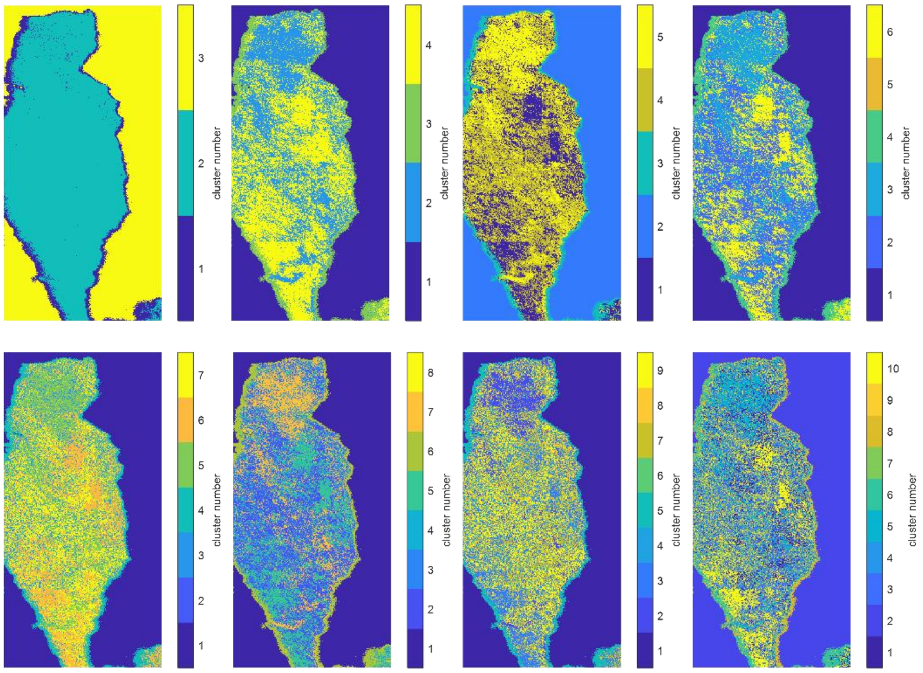

Figure S21: K-means images (K = 3-10) for DLBCL tissue 2.

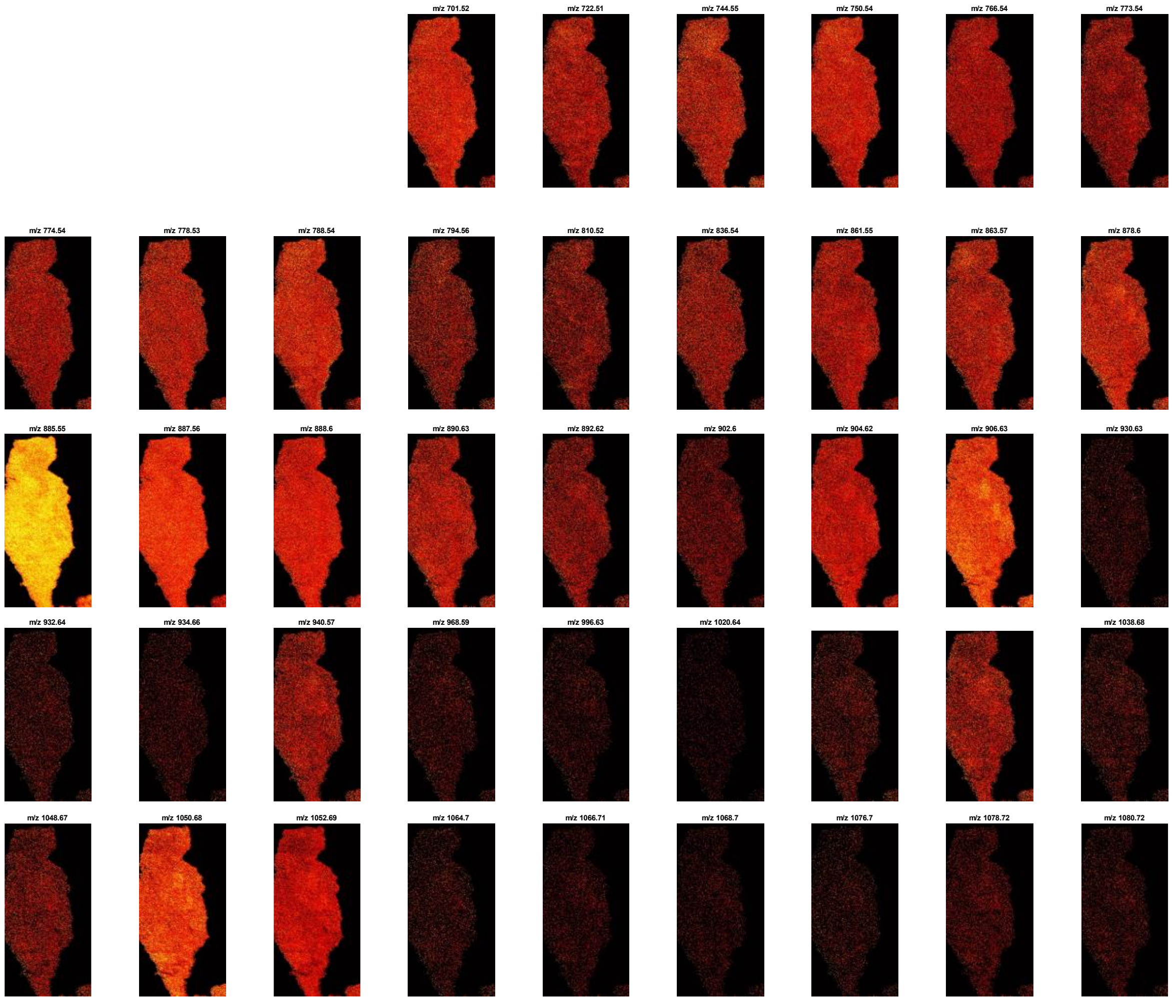

Figure S22: Ion images for DLBCL tissue 2.

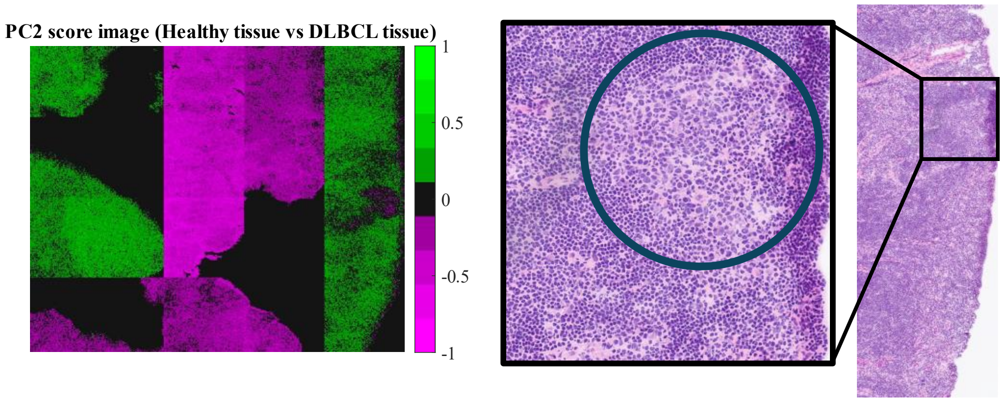

Figure S23. Misclassified region of tissue H7 in PC2 score image cut from Fig. 1a (left) and correlated H&E-stained image (right).

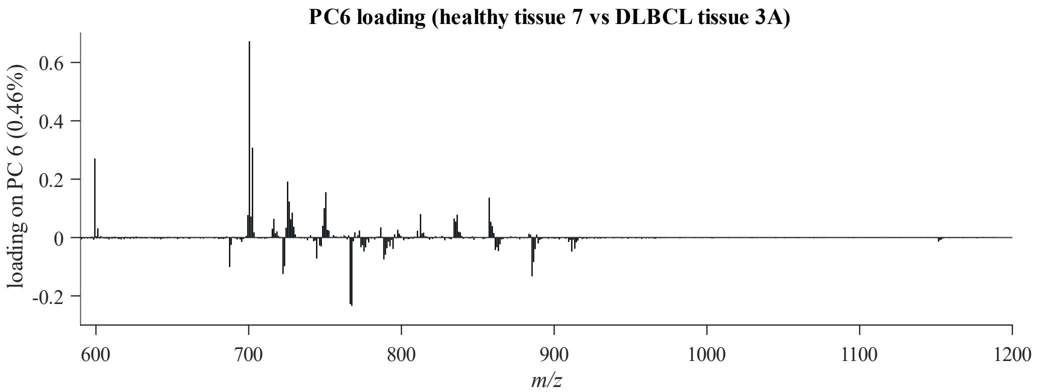

Figure S24: Full range of PC6 loading (healthy tissue 7 vs DLBCL tissue 3A).

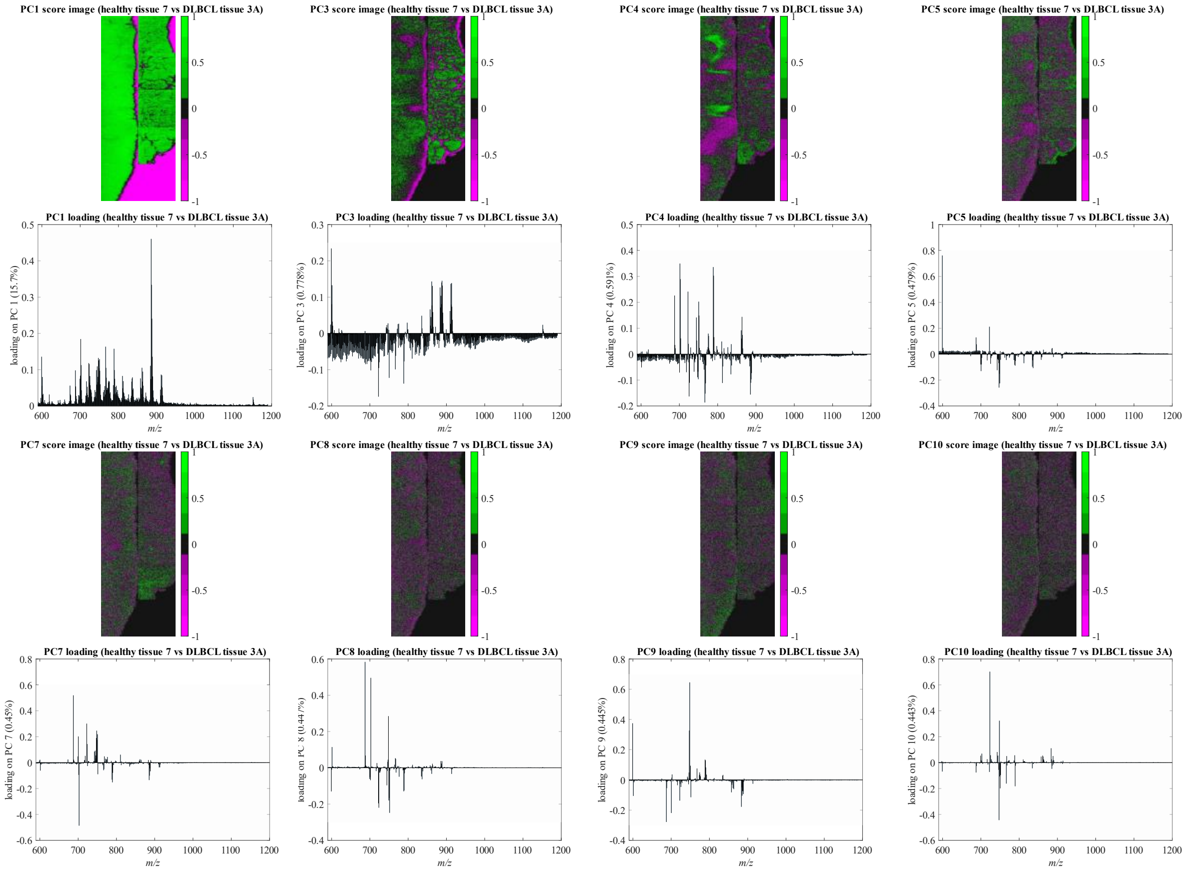

Figure S25: PCA results for healthy tissue 7 and DLBCL tissue 3A, PC 1, 2-5, and 7-10.

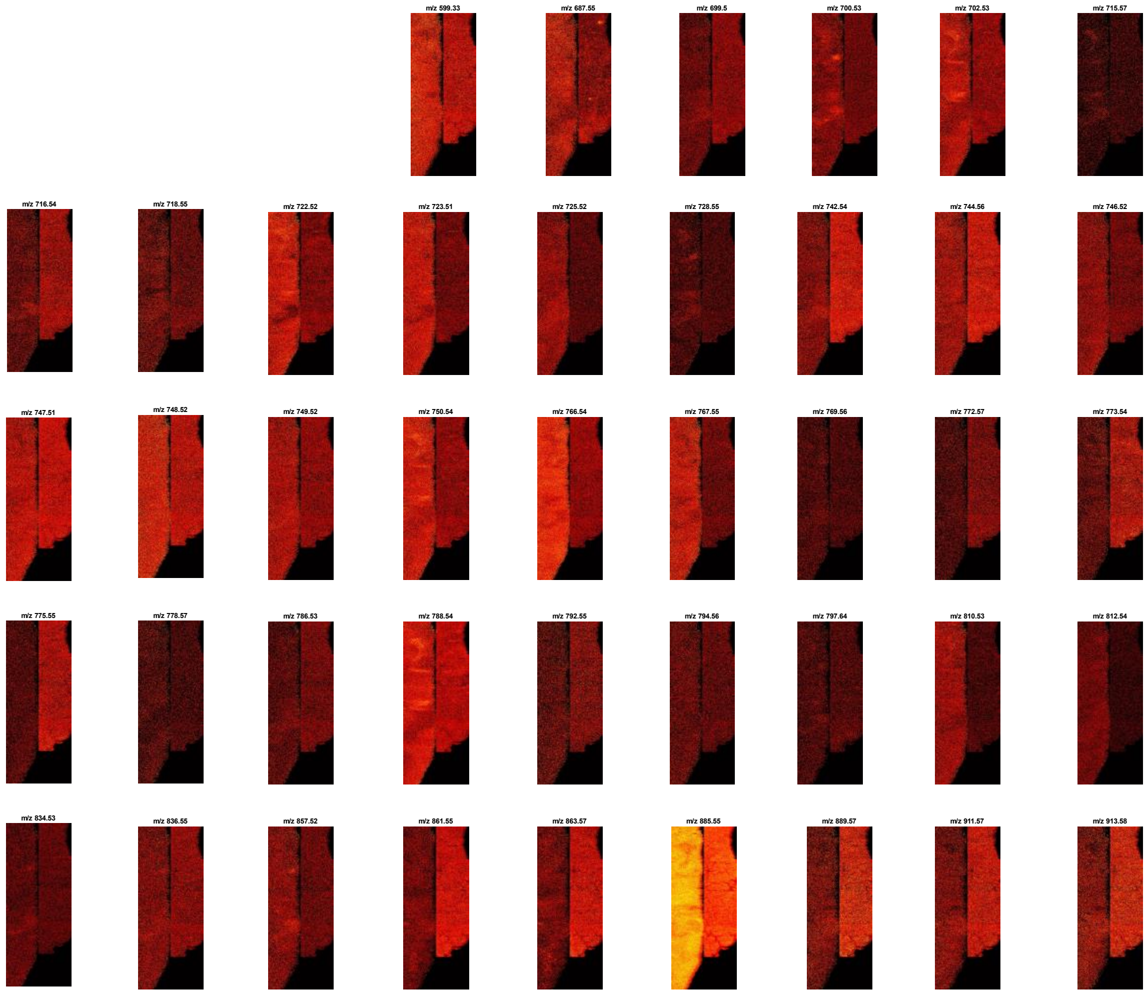

Figure S26: Ion images for healthy tissue 7 and DLBCL tissue 3A.

Supplemental tables

Table S1: Extended peak table of putatively identified lipids.

Detected *m/z*	Calculated *m/z*	Putatively identified lipid	Formula	Ion	Accuracy ppm	note	Metabolite ID level
1151.7165	1151.7048	NeuAcHex2Cer (34:1;O2)	$C_{57}H_{104}N_2O_{21}$	$[M-H]^-$	10.2		
1078.7166	1078.707	SHex2Cer (44:2)	$C_{56}H_{105}NO_{16}S$	-	8.9	Possible fragment ion	2-3
1052.6948	1052.6914	SHex2Cer (42:1)	$C_{54}H_{103}NO_{16}S$	-	3.2	Possible fragment ion	2-3
1050.6784	1050.6757	SHex2Cer (42:2)	$C_{54}H_{101}NO_{16}S$	-	2.6	Possible fragment ion	2-3
1048.6664	1048.6601	SHex2Cer (42:3)	$C_{54}H_{99}NO_{16}S$	-	6.0	Possible fragment ion	2-3
1024.6637	1024.6601	SHex2Cer (40:1)	$C_{52}H_{99}NO_{16}S$	-	3.5	Possible fragment ion	2-3
996.6288	996.6288	SHex2Cer (38:1)	$C_{50}H_{95}NO_{16}S$	-	0.0	Possible fragment ion	2-3
968.5931	968.5975	SHex2Cer (36:1)	$C_{48}H_{91}NO_{16}S$	-	-4.5	Possible fragment ion	2-3
940.5672	940.5662	SHex2Cer (34:1)	$C_{46}H_{87}NO_{16}S$	-	1.1	Possible fragment ion	2-3
915.5881	915.5957	PI (40:3)	$C_{49}H_{89}O_{13}P$	$[M-H]^-$	-8.3		2
913.5791	913.5801	PI (40:4)	$C_{49}H_{87}O_{13}P$	$[M-H]^-$	-1.1		2
911.569	911.5644	PI (40:5)	$C_{49}H_{85}O_{13}P$	$[M-H]^-$	5.0		2
909.5501	909.5488	PI (40:6)	$C_{49}H_{83}O_{13}P$	$[M-H]^-$	1.4		2
906.6321	906.6335	SHex2Cer (42:1) (OH)	$C_{48}H_{93}NO_{12}S$	-	-1.5	Possible fragment ion	2-3
904.6173	904.6178	SHexCer (42:2) (OH)	$C_{48}H_{91}NO_{12}S$	-	-0.6	Possible fragment ion	2-3
902.6045	902.6022	SHexCer (42:3) (OH)	$C_{48}H_{89}NO_{12}S$	-	2.5	Possible fragment ion	2-3
892.6208	892.6178	SHexCer (41:1) (OH)	$C_{47}H_{91}NO_{12}S$	-	3.4	Possible fragment ion	2-3
890.6324	890.6386	SHexCer (42:1)	$C_{48}H_{93}NO_{11}S$	-	-7.0	Possible fragment ion	2-3
889.5754	889.5801	PI (38:2)	$C_{47}H_{87}O_{13}P$	$[M-H]^-$	-5.3		2
887.561	887.5644	PI (38:3)	$C_{47}H_{85}O_{13}P$	$[M-H]^-$	-3.8		2
885.5462	885.5488	PI (38:4)	$C_{47}H_{83}O_{13}P$	$[M-H]^-$	-2.9	Calibration peak	2
878.5991	878.6022	SHexCer (40:1) (OH)	$C_{46}H_{89}NO_{12}S$	-	-3.5	Possible fragment ion	2-3
863.5666	863.5644	PI (36:1)	$C_{45}H_{85}O_{13}P$	$[M-H]^-$	2.5		2
861.5479	861.5488	PI (36:2)	$C_{45}H_{83}O_{13}P$	$[M-H]^-$	-1.0		2
859.5296	859.5331	PI (36:3)	$C_{45}H_{81}O_{13}P$	$[M-H]^-$	-4.1		2
857.5177	857.5175	PI (36:4)	$C_{45}H_{79}O_{13}P$	$[M-H]^-$	0.2		2
836.547	836.5436	PS (40:5)	$C_{46}H_{80}NO_{10}P$	$[M-H]^-$	4.1		2
835.5391	835.5331	PI (34:1)	$C_{43}H_{81}O_{13}P$	$[M-H]^-$	7.2		2
834.5291	834.528	PS (40:6)	$C_{46}H_{78}NO_{10}P$	$[M-H]^-$	1.3		2
812.5465	812.5436	PS (38:3)	$C_{44}H_{80}NO_{10}P$	$[M-H]^-$	3.6		2
810.5333	810.528	PS (38:4)	$C_{44}H_{78}NO_{10}P$	$[M-H]^-$	6.5		2
797.6352	797.6531	SM (42:2)	$C_{47}H_{93}N_2O_6P$	$[M-CH_3]^-$	-22.4		2
788.5416	788.5436	PS (36:1)	$C_{42}H_{80}NO_{10}P$	$[M-H]^-$	-2.5		2
786.5338	786.528	PS (36:2)	$C_{42}H_{78}NO_{10}P$	$[M-H]^-$	7.4		2
778.5735	778.5745	PE (O-40:5)	$C_{45}H_{82}NO_7P$	$[M-H]^-$	-1.3		2

775.5459	775.5484	PG (36:1)	$C_{42}H_{81}O_{10}P$	$[M-H]^-$	-3.2		2
774.5461	774.5432	PE (O-40:7)	$C_{45}H_{78}NO_7P$	$[M-H]^-$	3.7		2
773.5367	773.5327	PG (36:2)	$C_{42}H_{79}O_{10}P$	$[M-H]^-$	5.2		2
769.5565							
766.5396	766.5381	PE (38:4)	$C_{43}H_{78}NO_8P$	$[M-H]^-$	2.0		2
750.5398	750.5432	PE (O-38:5)	$C_{43}H_{78}NO_7P$	$[M-H]^-$	-4.5		2
749.5243	749.5116	PS (40:5)	$C_{46}H_{80}NO_{10}P$	$[M-H-C_3H_5O_2N]^-$	16.9	PS fragment ion	2
747.5131	747.5171	PG (34:1)	$C_{40}H_{77}O_{10}P$	$[M-H]^-$	-5.4		2
744.5522	744.5538	PE (36:1)	$C_{41}H_{80}NO_8P$	$[M-H]^-$	-2.1		2
742.5374	742.5381	PE (36:2)	$C_{41}H_{78}NO_8P$	$[M-H]^-$	-0.9	Calibration peak	2
740.5215	740.5225	PE (36:3)	$C_{41}H_{76}NO_8P$	$[M-H]^-$	-1.4		2
738.5087	738.5068	PE (36:4)	$C_{41}H_{74}NO_8P$	$[M-H]^-$	2.6		2
728.5525	728.5589	PE (O-36:2)	$C_{41}H_{80}NO_7P$	$[M-H]^-$	-8.8		2
725.5155	725.5198	PS (38:3)	$C_{41}H_{74}O_{10}$	$[M-H-C_3H_5O_2N]^-$	-5.9	PS fragment ion	2
723.5036	723.5042	PS (38:4)	$C_{41}H_{72}O_{10}$	$[M-H-C_3H_5O_2N]^-$	-0.8	PS fragment ion	2
722.5116	722.5119	PE (O-36:5)	$C_{41}H_{74}NO_7P$	$[M-H]^-$	-0.4		2
716.5358	716.5225	PE (34:1)	$C_{39}H_{76}NO_8P$	$[M-H]^-$	18.6		2
715.5654	715.5636	PA (O-38:1)	$C_{41}H_{81}O_7P$	$[M-H]^-$	2.5		2
714.5101	714.5068	PE (34:2)	$C_{39}H_{74}NO_8P$	$[M-H]^-$	4.6		2
702.5277	702.5432	PE (O-34:1)	$C_{39}H_{78}NO_7P$	$[M-H]^-$	-22.1		2
700.5312	700.5276	PE (O-34:2)	$C_{39}H_{76}NO_7P$	$[M-H]^-$	5.1		2
699.4987	699.4959	PS (36:2)	$C_{39}H_{73}O_8P$	$[M-H-C_3H_5O_2N]^-$	4.0	PS fragment ion	2
687.5493	687.5436	SM (34:1)	$C_{39}H_{79}N_2O_6P$	$[M-CH_3]^-$	8.3		2
619.2839	619.2878	LPI (20:4)	$C_{29}H_{49}O_{12}P$	-	-6.3	Fragment ion	2
599.3183	599.3191	LPI (18:0)	$C_{27}H_{53}O_{12}P$	-	-1.3	Fragment ion Calibration peak	2

Table S2: Complete readout from lipid maps with accepted assignments highlighted in yellow. Assignments highlighted in green were reassigned as known fragment ions of equivalent mass. Where no realistic assignment was provided by lipid maps (within a 0.01 m/z tolerance) assignments from lymphoma MS literature were used as indicated.

Input Mass	Matched Mass	Delta	Name	Formula	Ion	LMSD Examples Available	Literature Alternative (Y/N) or fragment
1151.7165	1151.7169	0.0004	PI 57:12;O	C66H105O14P	[M-H]-	N	Y
1151.7165	1151.7146	0.0019	PIP 50:3;O	C59H110O17P2	[M-H]-	N	
1078.7166	1078.7177	0.0011	MIPC 44:2;O2	C56H106NO16P	[M-H]-	N	Y
1078.7166	1078.7118	0.0048	PS 57:11;O	C63H102NO11P	[M-H]-	N	
1078.7166	1078.7118	0.0048	PT 56:11;O	C63H102NO10P	[M-H]-	N	
1078.7166	1078.7259	0.0093	Hex(3)-Cer 38:1;O2	C56H105NO18	[M-H]-	Y	
1052.6948	1052.6961	0.0013	PS 55:10;O	C61H100NO11P	[M-H]-	N	Y
1052.6948	1052.6961	0.0013	PT 54:10;O	C61H100NO10P	[M-H]-	N	
1052.6948	1052.6891	0.0057	Hex2Cer 45:6;O5	C57H99NO16	[M-H]-	N	
1052.6948	1052.7020	0.0072	MIPC 42:1;O2	C54H104NO16P	[M-H]-	N	
1050.6784	1050.6805	0.0021	PS 55:11;O	C61H98NO11P	[M-H]-	N	Y
1050.6784	1050.6805	0.0021	PT 54:11;O	C61H98NO10P	[M-H]-	N	
1050.6784	1050.6864	0.0080	MIPC 42:2;O2	C54H102NO16P	[M-H]-	N	
1048.6664	1048.6648	0.0016	PS 55:12;O	C61H96NO11P	[M-H]-	N	Y
1048.6664	1048.6648	0.0016	PT 54:12;O	C61H96NO10P	[M-H]-	N	
1048.6664	1048.6707	0.0043	MIPC 42:3;O2	C54H100NO16P	[M-H]-	N	
1024.6637	1024.6648	0.0011	PS 53:10;O	C59H96NO11P	[M-H]-	N	Y
1024.6637	1024.6648	0.0011	PT 52:10;O	C59H96NO10P	[M-H]-	N	
1024.6637	1024.6578	0.0059	Hex2Cer 43:6;O5	C55H95NO16	[M-H]-	N	
1024.6637	1024.6707	0.0070	MIPC 40:1;O2	C52H100NO16P	[M-H]-	N	
996.6288	996.6265	0.0023	Hex2Cer 41:6;O5	C53H91NO16	[M-H]-	N	Y
996.6288	996.6335	0.0047	PS 51:10;O	C57H92NO11P	[M-H]-	N	
996.6288	996.6335	0.0047	PT 50:10;O	C57H92NO10P	[M-H]-	N	
968.5931	968.5952	0.0021	Hex2Cer 39:6;O5	C51H87NO16	[M-H]-	N	Y
968.5931	968.6022	0.0091	PS 49:10;O	C55H88NO11P	[M-H]-	N	
968.5931	968.6022	0.0091	PT 48:10;O	C55H88NO10P	[M-H]-	N	
940.5672	940.5639	0.0033	Hex2Cer 37:6;O5	C49H83NO16	[M-H]-	N	Y
940.5672	940.5709	0.0037	PS 47:10;O	C53H84NO11P	[M-H]-	N	
940.5672	940.5709	0.0037	PT 46:10;O	C53H84NO10P	[M-H]-	N	
940.5672	940.5768	0.0096	MIPC 34:1;O2	C46H88NO16P	[M-H]-	N	
915.5881	915.5968	0.0087	PI 40:3	C49H89O13P	[M-H]-	Y	

915.5881	915.5968	0.0087	PI O-40:4;O	C49H89O13P	[M-H]-	N	
913.5791	913.5812	0.0021	PI 40:4	C49H87O13P	[M-H]-	Y	
913.5791	913.5812	0.0021	PI O-40:5;O	C49H87O13P	[M-H]-	N	
911.5690	911.5655	0.0035	PI 40:5	C49H85O13P	[M-H]-	Y	
911.5690	911.5655	0.0035	PI O-40:6;O	C49H85O13P	[M-H]-	N	
911.5690	911.5738	0.0048	DGDG 34:4	C49H84O15	[M-H]-	N	
909.5501	909.5499	0.0002	PI 40:6	C49H83O13P	[M-H]-	Y	
909.5501	909.5499	0.0002	PI O-40:7;O	C49H83O13P	[M-H]-	N	
909.5501	909.5581	0.0080	DGDG 34:5	C49H82O15	[M-H]-	N	
906.6321	906.6312	0.0009	HexCer 45:6;O6	C51H89NO12	[M-H]-	N	
906.6321	906.6346	0.0025	SHexCer 42:1;O3	C48H93NO12S	[M-H]-	Y	
906.6321	906.6382	0.0061	PE O-50:11	C55H90NO7P	[M-H]-	N	
906.6321	906.6230	0.0091	PS 45:5	C51H90NO10P	[M-H]-	N	
906.6321	906.6230	0.0091	PS O-45:6;O	C51H90NO10P	[M-H]-	N	
906.6321	906.6230	0.0091	PT 44:5	C51H90NO10P	[M-H]-	N	
906.6321	906.6230	0.0091	PT O-44:6;O	C51H90NO10P	[M-H]-	N	
906.6321	906.6382	0.0061	PC O-48:11	C56H92NO7P	[M-CH3]-	N	
904.6173	904.6189	0.0016	SHexCer 42:2;O3	C48H91NO12S	[M-H]-	Y	Y
904.6173	904.6226	0.0053	PE O-50:12	C55H88NO7P	[M-H]-	N	
904.6173	904.6073	0.0100	PS 45:6	C51H88NO10P	[M-H]-	N	
904.6173	904.6073	0.0100	PS O-45:7;O	C51H88NO10P	[M-H]-	N	
904.6173	904.6073	0.0100	PT 44:6	C51H88NO10P	[M-H]-	N	
904.6173	904.6073	0.0100	PT O-44:7;O	C51H88NO10P	[M-H]-	N	
904.6173	904.6226	0.0053	PC O-48:12	C56H90NO7P	[M-CH3]-	N	
902.6045	902.6033	0.0012	SHexCer 42:3;O3	C48H89NO12S	[M-H]-	N	
902.6045	902.6128	0.0083	IPC 42:3;O3	C48H90NO12P	[M-H]-	N	
892.6208	892.6226	0.0018	PE O-49:11	C54H88NO7P	[M-H]-	N	Y
892.6208	892.6189	0.0019	SHexCer 41:1;O3	C47H91NO12S	[M-H]-	N	
892.6208	892.6156	0.0052	HexCer 44:6;O6	C50H87NO12	[M-H]-	N	
892.6208	892.6284	0.0076	IPC 41:1;O3	C47H92NO12P	[M-H]-	N	
892.6208	892.6226	0.0018	PC O-47:11	C55H90NO7P	[M-CH3]-	N	
890.6324	890.6363	0.0039	HexCer 45:6;O5	C51H89NO11	[M-H]-	N	Y
890.6324	890.6281	0.0043	PE 46:6;O	C51H90NO9P	[M-H]-	N	
890.6324	890.6281	0.0043	PS O-45:6	C51H90NO9P	[M-H]-	N	
890.6324	890.6281	0.0043	PT O-44:6	C51H90NO10P	[M-H]-	N	
890.6324	890.6397	0.0073	SHexCer 42:1;O2	C48H93NO11S	[M-H]-	Y	
890.6324	890.6281	0.0043	PC 44:6;O	C52H92NO9P	[M-CH3]-	N	
889.5754	889.5753	0.0001	PA 51:12	C54H83O8P	[M-H]-	N	
889.5754	889.5753	0.0001	PEth 49:12	C54H83O8P	[M-H]-	N	

889.5754	889.5812	0.0058	PI 38:2	C47H87O13P	[M-H]-	Y	
889.5754	889.5812	0.0058	PI O-38:3;O	C47H87O13P	[M-H]-	N	
889.5754	889.5835	0.0081	MGDG 45:11	C54H82O10	[M-H]-	N	
887.5610	887.5655	0.0045	PI 38:3	C47H85O13P	[M-H]-	Y	
887.5610	887.5655	0.0045	PI O-38:4;O	C47H85O13P	[M-H]-	N	
887.5610	887.5679	0.0069	MGDG 45:12	C54H80O10	[M-H]-	N	
885.5462	885.5499	0.0037	PI 38:4	C47H83O13P	[M-H]-	Y	
885.5462	885.5499	0.0037	PI O-38:5;O	C47H83O13P	[M-H]-	N	
878.5991	878.5999	0.0008	HexCer 43:6;O6	C49H85NO12	[M-H]-		
878.5991	878.6033	0.0042	SHexCer 40:1;O3	C46H89NO12S	[M-H]-	Y	
878.5991	878.5917	0.0074	PS 43:5	C49H86NO10P	[M-H]-	N	
878.5991	878.5917	0.0074	PS O-43:6;O	C49H86NO10P	[M-H]-	N	
878.5991	878.5917	0.0074	PT 42:5	C49H86NO10P	[M-H]-	N	
878.5991	878.5917	0.0074	PT O-42:6;O	C49H86NO10P	[M-H]-	N	
878.5991	878.6069	0.0078	PE O-48:11	C53H86NO7P	[M-H]-	N	
878.5991	878.6069	0.0078	PC O-46:11	C54H88NO7P	[M-CH3]-	N	
863.5666	863.5655	0.0011	PI 36:1	C45H85O13P	[M-H]-	Y	
863.5666	863.5655	0.0011	PI O-36:2;O	C45H85O13P	[M-H]-	N	
863.5666	863.5679	0.0013	MGDG 43:10	C52H80O10	[M-H]-	N	
863.5666	863.5596	0.0070	PA 49:11	C52H81O8P	[M-H]-	N	
863.5666	863.5596	0.0070	PA O-49:12;O	C52H81O8P	[M-H]-	N	
863.5666	863.5596	0.0070	PEth 47:11	C52H81O8P	[M-H]-	N	
863.5666	863.5738	0.0072	DGDG 30:0	C45H84O15	[M-H]-	Y	
861.5479	861.5499	0.0020	PI 36:2	C45H83O13P	[M-H]-	Y	
861.5479	861.5499	0.0020	PI O-36:3;O	C45H83O13P	[M-H]-	N	
861.5479	861.5440	0.0039	PA 49:12	C52H79O8P	[M-H]-	N	
861.5479	861.5440	0.0039	PEth 47:12	C52H79O8P	[M-H]-	N	
861.5479	861.5522	0.0043	MGDG 43:11	C52H78O10	[M-H]-	N	
859.5296	859.5342	0.0046	PI 36:3	C45H81O13P	[M-H]-	Y	
859.5296	859.5342	0.0046	PI O-36:4;O	C45H81O13P	[M-H]-	N	
859.5296	859.5366	0.0070	MGDG 43:12	C52H76O10	[M-H]-	N	
857.5177	857.5186	0.0009	PI 36:4	C45H79O13P	[M-H]-	Y	
857.5177	857.5186	0.0009	PI O-36:5;O	C45H79O13P	[M-H]-	N	
857.5177	857.5268	0.0091	DGDG 30:3	C45H78O15	[M-H]-	N	
836.5470	836.5447	0.0023	PS 40:5	C46H80NO10P	[M-H]-	Y	
836.5470	836.5447	0.0023	PS O-40:6;O	C46H80NO10P	[M-H]-	N	
836.5470	836.5447	0.0023	PT 39:5	C46H80NO10P	[M-H]-	N	
836.5470	836.5447	0.0023	PT O-39:6;O	C46H80NO10P	[M-H]-	N	
836.5470	836.5530	0.0060	HexCer 40:6;O6	C46H79NO12	[M-H]-	N	
836.5470	836.5377	0.0093	Hex2Cer 30:1;O4	C42H79NO15	[M-H]-	N	

836.5470	836.5563	0.0093	SHexCer 37:1;O3	C43H83NO12S	[M-H]-	N	
835.5391	835.5366	0.0025	MGDG 41:10	C50H76O10	[M-H]-	N	
835.5391	835.5425	0.0034	DGDG 28:0	C43H80O15	[M-H]-	N	
835.5391	835.5342	0.0049	LPI 34:2;O	C43H81O13P	[M-H]-	N	
835.5391	835.5342	0.0049	PI 34:1	C43H81O13P	[M-H]-	Y	
835.5391	835.5342	0.0049	PI O-34:2;O	C43H81O13P	[M-H]-	N	
834.5291	834.5291	0.0000	PS 40:6	C46H78NO10P	[M-H]-	Y	
834.5291	834.5291	0.0000	PS O-40:7;O	C46H78NO10P	[M-H]-	N	
834.5291	834.5291	0.0000	PT 39:6	C46H78NO10P	[M-H]-	N	
834.5291	834.5291	0.0000	PT O-39:7;O	C46H78NO10P	[M-H]-	N	
834.5291	834.5221	0.0070	Hex2Cer 30:2;O4	C42H77NO15	[M-H]-	N	
812.5465	812.5447	0.0018	CerP 44:5;O6	C44H80NO10P	[M-H]-	N	
812.5465	812.5447	0.0018	PS 38:3	C44H80NO10P	[M-H]-	Y	
812.5465	812.5447	0.0018	PS O-38:4;O	C44H80NO10P	[M-H]-	N	
812.5465	812.5447	0.0018	PT 37:3	C44H80NO10P	[M-H]-	N	
812.5465	812.5447	0.0018	PT O-37:4;O	C44H80NO10P	[M-H]-	N	
812.5465	812.5530	0.0065	HexCer 38:4;O6	C44H79NO12	[M-H]-	N	
810.5333	810.5373	0.0040	HexCer 38:5;O6	C44H77NO12	[M-H]-	N	
810.5333	810.5291	0.0042	CerP 44:6;O6	C44H78NO10P	[M-H]-	N	
810.5333	810.5291	0.0042	PS 38:4	C44H78NO10P	[M-H]-	Y	
810.5333	810.5291	0.0042	PS O-38:5;O	C44H78NO10P	[M-H]-	N	
810.5333	810.5291	0.0042	PT 37:4	C44H78NO10P	[M-H]-	N	
810.5333	810.5291	0.0042	PT O-37:5;O	C44H78NO10P	[M-H]-	N	
810.5333	810.5407	0.0074	SHexCer 35:0;O3	C41H81NO12S	[M-H]-	N	
797.6352	797.6301	0.0051	DG 47:6;O2	C50H86O7	[M-H]-	N	Y
797.6352	797.6301	0.0051	TG 47:5;O	C50H86O7	[M-H]-	N	
797.6352	797.6301	0.0051	TG O-47:6;O2	C50H86O7	[M-H]-	N	
797.6352	797.6430	0.0078	PA O-44:2	C47H91O7P	[M-H]-	N	
788.5416	788.5447	0.0031	CerP 42:3;O6	C42H80NO10P	[M-H]-	N	
788.5416	788.5447	0.0031	PS 36:1	C42H80NO10P	[M-H]-	Y	
788.5416	788.5447	0.0031	PS O-36:2;O	C42H80NO10P	[M-H]-	N	
788.5416	788.5447	0.0031	PT 35:1	C42H80NO10P	[M-H]-	N	
788.5416	788.5447	0.0031	PT O-35:2;O	C42H80NO10P	[M-H]-	N	
788.5416	788.5471	0.0055	DGTA 39:10	C49H75NO7	[M-H]-	N	
788.5416	788.5471	0.0055	DGTS 39:10	C49H75NO7	[M-H]-	N	
786.5338	786.5314	0.0024	DGTA 39:11	C49H73NO7	[M-H]-	N	
786.5338	786.5314	0.0024	DGTS 39:11	C49H73NO7	[M-H]-	N	
786.5338	786.5373	0.0035	HexCer 36:3;O6	C42H77NO12	[M-H]-	N	
786.5338	786.5291	0.0047	CerP 42:4;O6	C42H78NO10P	[M-H]-	N	
786.5338	786.5291	0.0047	PS 36:2	C42H78NO10P	[M-H]-	Y	
786.5338	786.5291	0.0047	PS O-36:3;O	C42H78NO10P	[M-H]-	N	

786.5338	786.5291	0.0047	PT 35:2	C42H78NO10P	[M-H]-	N	
786.5338	786.5291	0.0047	PT O-35:3;O	C42H78NO10P	[M-H]-	N	
778.5735	778.5756	0.0021	CerP 45:5;O3	C45H82NO7P	[M-H]-	N	
778.5735	778.5756	0.0021	PE O-40:5	C45H82NO7P	[M-H]-	Y	
778.5735	778.5686	0.0049	HexCer 35:0;O6	C41H81NO12	[M-H]-	N	
778.5735	778.5756	0.0021	PC O-38:5	C46H84NO7P	[M-CH3]-	Y	
775.5459	775.5495	0.0036	PG 36:1	C42H81O10P	[M-H]-	Y	
775.5459	775.5495	0.0036	PG O-36:2;O	C42H81O10P	[M-H]-	N	
775.5459	775.5518	0.0059	DG 46:10;O2	C49H76O7	[M-H]-	N	
775.5459	775.5518	0.0059	TG 46:9;O	C49H76O7	[M-H]-	N	
775.5459	775.5518	0.0059	TG O-46:10;O2	C49H76O7	[M-H]-	N	
775.5459	775.5366	0.0093	MGDG 36:5	C45H76O10	[M-H]-	Y	
774.5461	774.5443	0.0018	PE O-40:7	C45H78NO7P	[M-H]-	Y	
774.5461	774.5526	0.0065	HexCer 39:6;O3	C45H77NO9	[M-H]-	N	
774.5461	774.5373	0.0088	HexCer 35:2;O6	C41H77NO12	[M-H]-	N	
774.5461	774.5443	0.0018	PC O-38:7	C46H80NO7P	[M-CH3]-	Y	
773.5367	773.5362	0.0005	DG 46:11;O2	C49H74O7	[M-H]-	N	
773.5367	773.5362	0.0005	TG 46:10;O	C49H74O7	[M-H]-	N	
773.5367	773.5362	0.0005	TG O-46:11;O2	C49H74O7	[M-H]-	N	
773.5367	773.5338	0.0029	PG 36:2	C42H79O10P	[M-H]-	Y	
773.5367	773.5338	0.0029	PG O-36:3;O	C42H79O10P	[M-H]-	N	
773.5367	773.5451	0.0084	CerPE 39:3;O5	C41H79N2O9P	[M-H]-	N	
773.5367	773.5451	0.0084	SM 37:3;O5	C42H81N2O9P	[M-CH3]-	N	
769.5565	769.5624	0.0059	TG 44:6;O2	C47H78O8	[M-H]-	N	N
769.5565	769.5624	0.0059	TG O-44:7;O3	C47H78O8	[M-H]-	N	
769.5565	769.5501	0.0064	CerPE 40:4;O4	C42H79N2O8P	[M-H]-	N	
769.5565	769.5501	0.0064	SM 38:4;O4	C43H81N2O8P	[M-CH3]-	N	
766.5396	766.5392	0.0004	CerP 43:5;O4	C43H78NO8P	[M-H]-	N	
766.5396	766.5392	0.0004	PE 38:4	C43H78NO8P	[M-H]-	Y	
766.5396	766.5392	0.0004	PE O-38:5;O	C43H78NO8P	[M-H]-	Y	
766.5396	766.5475	0.0079	HexCer 37:4;O4	C43H77NO10	[M-H]-	N	
766.5396	766.5392	0.0004	PC 36:4	C44H80NO8P	[M-CH3]-	Y	
766.5396	766.5392	0.0004	PC O-36:5;O	C44H80NO8P	[M-CH3]-	N	
750.5398	750.5373	0.0025	HexCer 33:0;O6	C39H77NO12	[M-H]-	N	
750.5398	750.5443	0.0045	CerP 43:5;O3	C43H78NO7P	[M-H]-	N	
750.5398	750.5443	0.0045	PE O-38:5	C43H78NO7P	[M-H]-	Y	
750.5398	750.5314	0.0084	DGTA 36:8	C46H73NO7	[M-H]-	N	
750.5398	750.5314	0.0084	DGTS 36:8	C46H73NO7	[M-H]-	N	
750.5398	750.5443	0.0045	PC O-36:5	C44H80NO7P	[M-CH3]-	Y	
749.5243	749.5239	0.0004	CerPE 40:6;O3	C42H75N2O7P	[M-H]-	N	PS fragment

749.5243	749.5209	0.0034	MGDG 34:4	C43H74O10	[M-H]-	Y	
749.5243	749.5338	0.0095	BMP 34:0	C40H79O10P	[M-H]-	N	
749.5243	749.5338	0.0095	LPG 34:1;O	C40H79O10P	[M-H]-	N	
749.5243	749.5338	0.0095	PG 34:0	C40H79O10P	[M-H]-	Y	
749.5243	749.5338	0.0095	PG O-34:1;O	C40H79O10P	[M-H]-	N	
749.5243	749.5239	0.0004	SM 38:6;O3	C43H77N2O7P	[M-CH3]-	N	
747.5131	747.5182	0.0051	BMP 34:1	C40H77O10P	[M-H]-	N	
747.5131	747.5182	0.0051	LPG 34:2;O	C40H77O10P	[M-H]-	N	
747.5131	747.5182	0.0051	PG 34:1	C40H77O10P	[M-H]-	Y	
747.5131	747.5182	0.0051	PG O-34:2;O	C40H77O10P	[M-H]-	N	
747.5131	747.5205	0.0074	DG 44:10;O2	C47H72O7	[M-H]-	N	
747.5131	747.5205	0.0074	TG 44:9;O	C47H72O7	[M-H]-	N	
747.5131	747.5205	0.0074	TG O-44:10;O2	C47H72O7	[M-H]-	N	
747.5131	747.5053	0.0078	MGDG 34:5	C43H72O10	[M-H]-	N	
744.5522	744.5549	0.0027	CerP 41:2;O4	C41H80NO8P	[M-H]-	N	
744.5522	744.5549	0.0027	LPC 33:2;O	C41H80NO8P	[M-H]-	N	
744.5522	744.5549	0.0027	LPT O-34:2	C41H80NO10P	[M-H]-	N	
744.5522	744.5549	0.0027	PE 36:1	C41H80NO8P	[M-H]-	Y	
744.5522	744.5549	0.0027	PE O-36:2;O	C41H80NO8P	[M-H]-	N	
744.5522	744.5549	0.0027	PC 34:1	C42H82NO8P	[M-CH3]-	Y	
744.5522	744.5549	0.0027	PC O-34:2;O	C42H82NO8P	[M-CH3]-	N	
742.5374	742.5392	0.0018	CerP 41:3;O4	C41H78NO8P	[M-H]-	N	
742.5374	742.5392	0.0018	LPC 33:3;O	C41H78NO8P	[M-H]-	N	
742.5374	742.5392	0.0018	LPT O-34:3	C41H78NO10P	[M-H]-	N	
742.5374	742.5392	0.0018	PE 36:2	C41H78NO8P	[M-H]-	Y	
742.5374	742.5392	0.0018	PE O-36:3;O	C41H78NO8P	[M-H]-	N	
742.5374	742.5392	0.0018	PC 34:2	C42H80NO8P	[M-CH3]-	Y	
742.5374	742.5392	0.0018	PC O-34:3;O	C42H80NO8P	[M-CH3]-	N	
740.5215	740.5236	0.0021	CerP 41:4;O4	C41H76NO8P	[M-H]-	N	
740.5215	740.5236	0.0021	LPC 33:4;O	C41H76NO8P	[M-H]-	N	
740.5215	740.5236	0.0021	LPT O-34:4	C41H76NO10P	[M-H]-	N	
740.5215	740.5236	0.0021	PE 36:3	C41H76NO8P	[M-H]-	Y	
740.5215	740.5236	0.0021	PE O-36:4;O	C41H76NO8P	[M-H]-	N	
740.5215	740.5236	0.0021	PC 34:3	C42H78NO8P	[M-CH3]-	Y	
740.5215	740.5236	0.0021	PC O-34:4;O	C42H78NO8P	[M-CH3]-	N	
738.5087	738.5079	0.0008	CerP 41:5;O4	C41H74NO8P	[M-H]-	N	
738.5087	738.5079	0.0008	LPC 33:5;O	C41H74NO8P	[M-H]-	N	
738.5087	738.5079	0.0008	LPT O-34:5	C41H74NO10P	[M-H]-	N	
738.5087	738.5079	0.0008	PE 36:4	C41H74NO8P	[M-H]-	Y	
738.5087	738.5079	0.0008	PE O-36:5;O	C41H74NO8P	[M-H]-	Y	
738.5087	738.5162	0.0075	HexCer 35:4;O4	C41H73NO10	[M-H]-	N	
738.5087	738.5079	0.0008	PC 34:4	C42H76NO8P	[M-CH3]-	Y	
738.5087	738.5079	0.0008	PC O-34:5;O	C42H76NO8P	[M-CH3]-	N	

728.5525	728.5471	0.0054	ACer 44:6;O5	C44H75NO7	[M-H]-	N	
728.5525	728.5471	0.0054	DGTA 34:5	C44H75NO7	[M-H]-	N	
728.5525	728.5471	0.0054	DGTS 34:5	C44H75NO7	[M-H]-	N	
728.5525	728.5600	0.0075	CerP 41:2;O3	C41H80NO7P	[M-H]-	N	
728.5525	728.5600	0.0075	LPC 33:2	C41H80NO7P	[M-H]-	N	
728.5525	728.5600	0.0075	LPC O-33:3;O	C41H80NO7P	[M-H]-	N	
728.5525	728.5600	0.0075	PE O-36:2	C41H80NO7P	[M-H]-	Y	
728.5525	728.5600	0.0075	PC O-34:2	C42H82NO7P	[M-CH3]-	Y	
725.5155	725.5151	0.0004	DG 45:12	C48H70O5	[M-H]-	N	
725.5155	725.5151	0.0004	TG O-45:12	C48H70O5	[M-H]-	N	
725.5155	725.5127	0.0028	PA 38:3	C41H75O8P	[M-H]-	Y	PS fragment
725.5155	725.5127	0.0028	PA O-38:4;O	C41H75O8P	[M-H]-	N	
725.5155	725.5127	0.0028	PEth 36:3	C41H75O8P	[M-H]-	Y	
725.5155	725.5209	0.0054	MGDG 32:2	C41H74O10	[M-H]-	Y	
725.5155	725.5087	0.0068	CerPE 34:0;O6	C36H75N2O10P	[M-H]-	N	
725.5155	725.5239	0.0084	CerPE 38:4;O3	C40H75N2O7P	[M-H]-	N	
725.5155	725.5087	0.0068	SM 32:0;O6	C37H77N2O10P	[M-CH3]-	N	
725.5155	725.5239	0.0084	SM 36:4;O3	C41H77N2O7P	[M-CH3]-	N	
723.5036	723.5053	0.0017	MGDG 32:3	C41H72O10	[M-H]-	Y	
723.5036	723.5083	0.0047	CerPE 38:5;O3	C40H73N2O7P	[M-H]-	N	
723.5036	723.4970	0.0066	PA 38:4	C41H73O8P	[M-H]-	Y	PS fragment
723.5036	723.4970	0.0066	PA O-38:5;O	C41H73O8P	[M-H]-	N	
723.5036	723.4970	0.0066	PEth 36:4	C41H73O8P	[M-H]-	Y	
723.5036	723.5083	0.0047	SM 36:5;O3	C41H75N2O7P	[M-CH3]-	N	
722.5116	722.5130	0.0014	CerP 41:5;O3	C41H74NO7P	[M-H]-	N	
722.5116	722.5130	0.0014	LPC 33:5	C41H74NO7P	[M-H]-	N	
722.5116	722.5130	0.0014	LPC O-33:6;O	C41H74NO7P	[M-H]-	N	
722.5116	722.5130	0.0014	PE O-36:5	C41H74NO7P	[M-H]-	Y	
722.5116	722.5060	0.0056	HexCer 31:0;O6	C37H73NO12	[M-H]-	N	
722.5116	722.5213	0.0097	HexCer 35:4;O3	C41H73NO9	[M-H]-	N	
722.5116	722.5130	0.0014	PC O-34:5	C42H76NO7P	[M-CH3]-	Y	
716.5358	716.5318	0.0040	HexCer 33:1;O4	C39H75NO10	[M-H]-	N	Y
715.5654	715.5647	0.0007	PA O-38:1	C41H81O7P	[M-H]-	Y	
715.5654	715.5671	0.0017	CE 21:5;O2	C48H76O4	[M-H]-	N	
715.5654	715.5671	0.0017	DG O-45:10	C48H76O4	[M-H]-	N	
714.5101	714.5079	0.0022	CerP 39:3;O4	C39H74NO8P	[M-H]-	N	
714.5101	714.5079	0.0022	LPC 31:3;O	C39H74NO8P	[M-H]-	N	
714.5101	714.5079	0.0022	LPE 34:3;O	C39H74NO8P	[M-H]-	N	
714.5101	714.5079	0.0022	LPS O-33:3	C39H74NO8P	[M-H]-	N	
714.5101	714.5079	0.0022	LPT O-32:3	C39H74NO10P	[M-H]-	N	
714.5101	714.5079	0.0022	PE 34:2	C39H74NO8P	[M-H]-	Y	

714.5101	714.5079	0.0022	PE O-34:3;O	C39H74NO8P	[M-H]-	N	
714.5101	714.5162	0.0061	HexCer 33:2;O4	C39H73NO10	[M-H]-	N	
714.5101	714.5079	0.0022	PC 32:2	C40H76NO8P	[M-CH3]-	Y	
714.5101	714.5079	0.0022	PC O-32:3;O	C40H76NO8P	[M-CH3]-	N	
702.5277	702.5314	0.0037	ACer 42:5;O5	C42H73NO7	[M-H]-	N	
702.5277	702.5314	0.0037	Cer 42:6;O6	C42H73NO7	[M-H]-	N	
702.5277	702.5314	0.0037	DGTA 32:4	C42H73NO7	[M-H]-	N	
702.5277	702.5314	0.0037	DGTS 32:4	C42H73NO7	[M-H]-	N	
700.5312	700.5287	0.0025	CerP 39:2;O3	C39H76NO7P	[M-H]-	N	
700.5312	700.5287	0.0025	LPC 31:2	C39H76NO7P	[M-H]-	N	
700.5312	700.5287	0.0025	LPC O-31:3;O	C39H76NO7P	[M-H]-	N	
700.5312	700.5287	0.0025	LPE 34:2	C39H76NO7P	[M-H]-	N	
700.5312	700.5287	0.0025	LPE O-34:3;O	C39H76NO7P	[M-H]-	N	
700.5312	700.5287	0.0025	PE O-34:2	C39H76NO7P	[M-H]-	Y	
700.5312	700.5369	0.0057	HexCer 33:1;O3	C39H75NO9	[M-H]-	N	
700.5312	700.5287	0.0025	PC O-32:2	C40H78NO7P	[M-CH3]-	Y	
699.4987	699.4994	0.0007	DG 43:11	C46H68O5	[M-H]-	N	
699.4987	699.4994	0.0007	DG O-43:12;O	C46H68O5	[M-H]-	N	
699.4987	699.4994	0.0007	TG O-43:11	C46H68O5	[M-H]-	N	
699.4987	699.4970	0.0017	LPG O-33:4	C39H73O8P	[M-H]-	N	
699.4987	699.4970	0.0017	PA 36:2	C39H73O8P	[M-H]-	Y	PS fragment
699.4987	699.4970	0.0017	PA O-36:3;O	C39H73O8P	[M-H]-	N	
699.4987	699.4970	0.0017	PEth 34:2	C39H73O8P	[M-H]-	Y	
699.4987	699.5053	0.0066	MGDG 30:1	C39H72O10	[M-H]-	Y	
699.4987	699.5083	0.0096	CerPE 36:3;O3	C38H73N2O7P	[M-H]-	Y	
699.4987	699.5083	0.0096	SM 34:3;O3	C39H75N2O7P	[M-CH3]-	N	
687.5493	687.5447	0.0046	CerPE 36:1;O2	C38H77N2O6P	[M-H]-	Y	
687.5493	687.5569	0.0076	CE 16:0;O4	C43H76O6	[M-H]-	N	
687.5493	687.5569	0.0076	DG 40:4;O	C43H76O6	[M-H]-	N	
687.5493	687.5569	0.0076	DG O-40:5;O2	C43H76O6	[M-H]-	N	
687.5493	687.5569	0.0076	TG 40:3	C43H76O6	[M-H]-	N	
687.5493	687.5569	0.0076	TG O-40:4;O	C43H76O6	[M-H]-	N	
687.5493	687.5447	0.0046	SM 34:1;O2	C39H79N2O6P	[M-CH3]-	Y	
619.2839	619.2794	0.0045	SQDG 20:3	C29H48O12S	[M-H]-	N	
619.2839	619.2889	0.0050	LPI 20:4	C29H49O12P	[M-H]-	Y	
619.2839	619.2889	0.0050	LPI O-20:5;O	C29H49O12P	[M-H]-	N	
619.2839	619.2889	0.0050	PI O-20:4	C29H49O12P	[M-H]-	N	
619.2839	619.2760	0.0079	ST 26:5;O6;GlcA	C32H44O12	[M-H]-	N	
619.2839	619.2760	0.0079	ST 26:6;O7;Hex	C32H44O12	[M-H]-	Y	
599.3183	599.3202	0.0019	LPI 18:0	C27H53O12P	[M-H]-	Y	
599.3183	599.3202	0.0019	LPI O-18:1;O	C27H53O12P	[M-H]-	N	
599.3183	599.3226	0.0043	ST 28:5;O3;GlcA	C34H48O9	[M-H]-	N	

599.3183	599.3226	0.0043	ST 28:6;O4;Hex	C34H48O9	[M-H]-	N	
599.3183	599.3226	0.0043	TG 31:8;O3	C34H48O9	[M-H]-	N	
